# Colorectal cancer-infiltrating NK cell landscape analysis unravels tissue-resident PD-1^+^ NK cells in microsatellite instability tumors

**DOI:** 10.3389/fimmu.2025.1578444

**Published:** 2025-06-18

**Authors:** Valentina Obino, Chiara Giordano, Simona Carlomagno, Chiara Setti, Marco Greppi, Matteo Bozzo, Silvia Pesce, Elisa Ferretti, Simona Candiani, Letizia Muccio, Enrico Ciferri, Tania Buttiron Webber, Agnese Solari, Fulvia Ortolani, Laura Paleari, Matteo Clavarezza, Andrea Barberis, Marco Filauro, Nicoletta Provinciali, Mariangela Rutigliani, Emanuela Marcenaro, Andrea DeCensi, Mariella Della Chiesa, Simona Sivori

**Affiliations:** ^1^ Department of Experimental Medicine (DIMES), University of Genoa, Genoa, Italy; ^2^ Department of Medicine (DMED), University of Udine, Udine, Italy; ^3^ Department of Earth, Environmental and Life Sciences (DISTAV), University of Genoa, Genoa, Italy; ^4^ Direzione Scientifica, IRCCS Ospedale Policlinico San Martino, Genoa, Italy; ^5^ U.O. Medicina di Laboratorio, IRCCS Ospedale Policlinico San Martino, Genoa, Italy; ^6^ Department of Abdominal Surgery – General and Hepatobiliopancreatic Surgery Unit, E.O. Ospedali Galliera, Genoa, Italy; ^7^ Division of Medical Oncology, E.O. Ospedali Galliera, Genoa, Italy; ^8^ ALiSa, Liguria Health Authority, Genoa, Italy; ^9^ Department of Laboratory and Service – Histological and Anatomical Pathology Unit, E.O. Ospedali Galliera, Genoa, Italy; ^10^ Wolfson Institute of Population Health, Queen Mary University of London, London, United Kingdom

**Keywords:** human NK cells, colorectal cancer, immune checkpoints, tissue-residency markers, microsatellite status

## Abstract

**Background:**

Natural killer (NK) cells are innate lymphocytes endowed with potent cytotoxic activity. The presence of tumor-associated NK cells has been correlated with better prognosis in several solid tumors including colorectal cancer (CRC). This malignant disease is the second cause of cancer death worldwide and is in urgent need for novel approaches to improve current immunotherapies. Since CRC microenvironment can induce NK cell dysfunction and hinder cancer control, understanding tumor-associated NK cell features is mandatory to fully unlock their immunotherapeutic potential.

**Purpose:**

Our study aims at elucidating the molecular and functional characteristics of tumor-associated NK cells in CRC focusing on the expression of immune checkpoints that critically regulate NK cell function. We performed an in-depth cytofluorimetric analysis of tumor-associated NK cells obtained by tissue dissociation of samples derived from 80 CRC patients comparing tumor with matched tumor-free tissue and peripheral blood, stratifying patients by tumor stage or MSI/MSS condition. Tumor tissue was also analyzed by immunohistochemistry.

**Results:**

NK cells expressing immune checkpoints (i.e., KIR, NKG2A and TIM-3) were significantly enriched in tumor compared to tumor-free tissue, and an increase in PD-1^+^ NK cells was observed in tumors compared to peripheral blood and tumor-free tissue, indicating TME-induced modulation. Notably, tumor-associated PD-1^+^ NK cells characterized MSI rather than MSS CRC. In addition, tumor-associated NK cells also expressed tissue residency markers (CD103 and/or CD49a) and displayed a distinct profile also including the PD-1^+^ NK cell subset in MSI CRC, possibly representing NK cells recruited from circulation, retained in tumors, and reconfigured by TME signals. Importantly, tissue resident NK cells adequately expressed activating NK receptors and cytotoxic molecules.

**Conclusions:**

These results suggest, together with an increased PD-L1 expression on MSI tumor cells, that the efficacy of immunotherapies in MSI CRC based on PD-1/PD-L1 blockade could also rely on a superior anti-tumor potential of PD-1^+^ NK cells. Conversely, MSS CRC, in which tumor-associated PD-1^+^ NK cells are scarce, could benefit more from immunotherapies blocking NKG2A and/or KIRs. Thus, novel approaches based on NK cell features related to CRC type, fully exploiting circulating and resident NK cell anti-tumor activity, could be key to next-generation therapies.

## 1 Introduction

The central role of NK cells in controlling tumor growth has been demonstrated in numerous preclinical studies concerning not only hematological malignancies but also solid tumors such as colorectal cancer (CRC) (1, 2).

NK cells are both “serial killers”, capable of killing multiple targets without requiring any prior antigen exposure, and efficient producers of cytokines important for regulating additional innate immune cells activity as well as for influencing adaptive immune responses (3–5). NK cells belong to a heterogeneous family of innate lymphoid cells (ILCs) also involved in host-defense and tissue repair, showing superior cytotoxic properties compared to other ILCs, which are generally identified by IL-7 receptor-α chain (CD127) expression and referred to as innate counterparts of T helper cells (“helper” ILC1s, ILC2s and ILC3s) (5, 6).

NK cell activity is finely regulated by activating and inhibitory receptors (4), the latter including the HLA class I (HLA-I) specific CD94/NKG2A heterodimer that recognizes the non-classical molecule HLA-E, and the inhibitory Killer-cell Immunoglobulin-like Receptors (iKIRs) that recognize epitopes of classical HLA-I molecules (HLA-A, -B to -C) (7). Besides HLA-I specific receptors that grant self-tolerance and recognition of pathological HLA-I alterations, NK cells can express other non-HLA-I specific inhibitory receptors that act as immune checkpoints (ICs) on NK cell function. These additional ICs include PD-1, TIM-3, TIGIT, LAG-3 and can be up-regulated and/or *de-novo* expressed in pathological conditions such as infections or under the influence of the tumor microenvironment (TME) (4, 8), weakening NK responses.

Most of our knowledge about NK cells relates to conventional NK cells (cNK) that develop in the bone marrow, populate the blood and then circulate from blood to tissues. However, recent studies are disclosing information about the understudied tissue-resident NK cells (trNK), found in some non-lymphoid tissues and whose developmental stages are poorly known (6, 9, 10). Indeed, increasing knowledge on trNK cells will help define their identity and possible relationship to circulating cNK cells in different pathological conditions including solid tumors.

CRC is a public health issue since it is the third high incidence cancer and the second most frequent cause of cancer-related deaths worldwide (11) with little therapeutic improvements in the last years, except for given CRC types.

The molecular mechanisms described in CRC carcinogenesis and progression include chromosomal instability (CIN), defects in DNA repair (mismatch repair deficiency, dMMR) and microsatellite instability (MSI), aberrant DNA hypermethylation, giving rise to a high heterogeneity of tumor genetic patterns and clinical outcomes (12). In particular, MSI CRC tumors result from the mutation in at least 1 of the 4 MMR genes (MLH1, MSH2, MSH6 and PMS2) (13) leading to a rapid accumulation of genetic errors and causing MSI. Because of their instability, MSI CRCs display a high tumor mutational burden (TMB) resulting in an increased production of neoantigens, which can induce abundance of tumor infiltrating lymphocytes (TIL) and their activation (14). In line with a high TIL presence, MSI condition is considered a good predictor of both CRC prognosis and greater sensitivity to IC blockade immunotherapies. In particular, the blockade of the PD-1/PD-Ls axis has been demonstrated to be highly effective in a group of MSI/dMMR CRC patients (15), whereas the efficacy of other immunotherapeutic approaches is variable (16), probably due to the high heterogeneity of CRCs. In this context, immune-mediated therapies aimed at increasing TILs function in tumor tissues could be a useful strategy in CRC containment. Interestingly, tumor-infiltrating NK cells have been associated with an improved CRC prognosis in both primary and metastatic disease (1, 17). However, the precise role of NK cells in the CRC TME remains poorly understood.

In this study, we performed an in-depth cytofluorimetric analysis of the expression of different ICs critically regulating NK cell function on tumor-associated NK cells, by applying a bona fide NK cell gating strategy excluding CD127^+^ ILCs. Specifically, tumor samples from 80 patients with CRC were analyzed in comparison with matched tumor-free tissue and peripheral blood, stratifying patients by tumor stage, aggregating early stages I-II vs advanced stages III-IV (as reported in previous studies (18)) or MSI/microsatellite stability (MSS) condition.

We also distinguished trNK cells from non-trNK cells, to better understand the possible contribution of specific NK cell subsets in IC blockade immunotherapy and suggest novel NK-based therapeutic approaches against CRC tumor and its metastases, exploiting the antitumor activity of circulating NK cells and, more importantly, of resident NK cell subsets within the TME. Our study showed that NK cells expressing immune checkpoints (including NKG2A and KIRs that typically inhibit NK cell function upon HLA-I recognition) are enriched in tumor tissues from CRC patients regardless of the tumor stage and MSI/MSS status. In contrast, tumor-associated trNK cells expressing PD-1 characterize only MSI-CRC, together with higher PD-L1^+^ tumor cells, suggesting that different NK-enhancing strategies are required in distinct CRC types.

## 2 Methods

### 2.1 Ethics statements, patients and tissue/peripheral blood samples

The study was conducted in accordance with the Declaration of Helsinki and was approved by the Ethics Committee of the Liguria Region, Genova, Italy (Galliera codex: 64UCS2018 for CRC patient cohort and n. 39/2012 number CER Liguria: DB id 10125 for healthy donors). All subjects gave written informed consent to participate in the study before taking part.

The cohort analyzed in this study includes 80 patients diagnosed with CRC, described in detail in **Table 1**, undergoing surgical excision of colorectal tumor at the Department of Abdominal Surgery-General Pancreatic and Hepatobiliary Surgery Unit, E.O. Galliera Hospital (Genoa, Italy). All patients enrolled had not received any neoadjuvant treatment before tumor resection. Pathological staging of all CRC samples was determined according to TNM classification of malignant tumors. For each patient, primary CRC tissue, colorectal healthy mucosa (referred to as tumor-free tissue, obtained from areas more than 2 cm away from the tumor site) and when possible, metastatic lymph nodes were collected and examined by the pathologist and immediately immersed in MACS^®^ Tissue Storage Solution (Miltenyi Biotec, Bergisch Gladbach, Germany). In addition to the surgical fragments, PB was also obtained from each patient immediately before surgery. PB from adult healthy donors (HD) aged over 40 years and under 65 years, were obtained from the Transfusion Center of the IRCCS Ospedale Policlinico San Martino (Genoa, Italy) and used as controls. PB Mononuclear cells (PBMCs) were isolated by density gradient separation with Lympholyte^®^-H (Cedarlane Labs, Burlington, Canada).

**Table 1:** Clinical characteristics of CRC patients.

Demographical and clinical data	n (%)
CRC patients	80 (100%)
Sex	
Male	40 (50%)
Female	40 (50%)
Age	
<65	13 (16,25%)
65-75	20 (25%)
>75	47 (58,75%)
Race	
Caucasian	78 (94,5%)
African	1 (1,25%)
Asian	1 (1,25%)
Tumor stage	
I	7 (8,75%)
II	39 (48,75%)
III	26 (32,5 %)
IV	8 (10%)
MMR status	
MMR deficient (MSI)	21 (26,25%)
MMR proficient (MSS)	59 (73,75%)

MMR, mismatch repair; MSI, microsatellite instability; MSS, microsatellite stability.

### 2.2 Tissue dissociation

After removal of necrotic and fat tissues, the surgical tissue fragments were weighted (weight range 1–4 g), cut into small pieces (<0.5 cm) and transferred into gentleMACS™ C Tubes (Miltenyi Biotec) (≤1 g/tube). Then, they were mechanically and enzymatically dissociated using the Tumor Dissociation Kit human (Miltenyi Biotec) and the gentleMACS™ Octo Dissociator with Heaters (Miltenyi Biotec) accordingly to manufacturers’ instructions. The resulting single cell suspension was filtered through a 100 μm-cell strainer (Miltenyi Biotec) to remove cellular and fibrous aggregates and washed twice with RPMI-1640 supplemented with 2mM EDTA (Sigma-Aldrich^®^, Merck Group, Darmstadt, Germany). Cells were then resuspended in a complete culture medium (RPMI-1640) supplemented with 2mM glutamine, 1% penicillin, 1% streptomycin and 10% heat-inactivated FCS (Gibco, ThermoFisher Scientific, Waltham, Massachusetts, USA), counted with trypan blue dye to exclude dead cells and used for cytofluorimetric analyses or frozen in liquid nitrogen for subsequent evaluations. Tissue sample size and/or yield were heterogeneous and limited in some cases, therefore some analyses were performed on a smaller number of samples, reported in figure legends.

### 2.3 Flow cytometry, monoclonal antibodies, degranulation assay, and MSI/MSS state assessment

Phenotypic analyses of surface and intracellular markers on NK cells from PBMCs and cell suspensions of tumor and tumor-free tissues were performed by multiparametric flow cytometry on an 18-colors LSR Fortessa flow cytometer (BD Biosciences, San Jose, CA, USA). Detailed methods for flow cytometry gating strategies/analyses, monoclonal antibodies, functional assays as well as immunohistochemistry procedures on tumor tissue sections are reported in **Supplementary Material**.

### 2.4 Statistical analysis

To compare the different cell subsets considered throughout this study, statistical analyses were performed using Kruskal-Wallis test followed by Dunn´s test for multiple comparisons, or multiple Mann-Whitney test with Holm-Sidak for multiple comparisons, or two-tailed Mann-Whitney U-test in GraphPad Prism version 8.0 (GraphPad Software, www.graphpad.com). Data were represented as mean ± standard deviation (SD) and p-values < 0.05 were considered statistically significant and shown as follows: * p<0.05, ** p<0.01, *** p<0.001 and **** p<0.0001.

## 3 Results

### 3.1 NK cells are similarly present in tumor and tumor-free tissues from CRC patients

**Figure 1:**
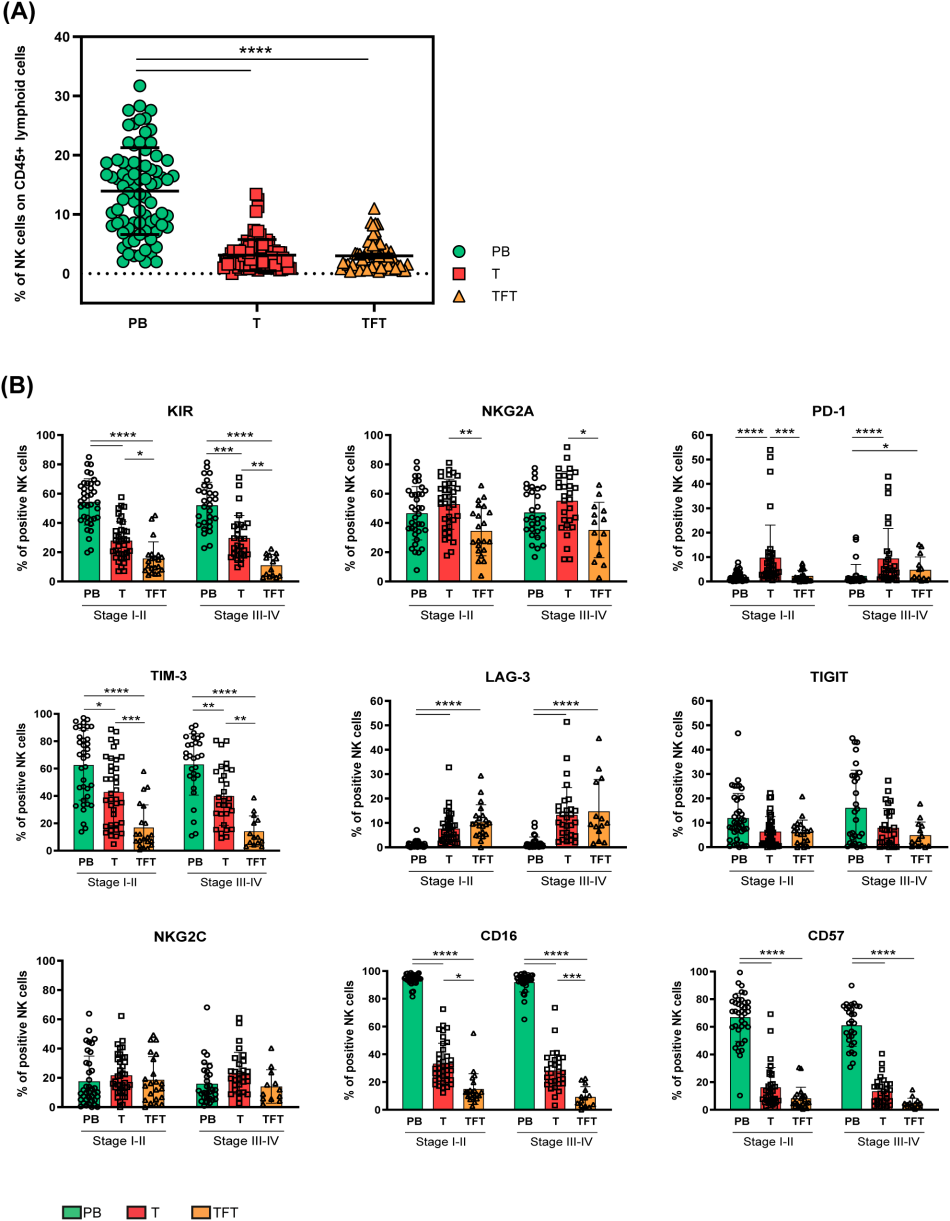
NK cell abundance and heterogeneity in peripheral blood, tumor and tumor-free tissues from CRC patients. **(A)** The frequency of NK cells among CD45^+^ lymphoid cells derived from peripheral blood (PB, green circles), tumor (T, red squares), and tumor-free tissue (TFT, orange triangles) of CRC patients is depicted as mean±SD (PB/T n=80, TFT n=58). Statistical significance calculated by Kruskal-Wallis test is indicated. (****p<0.0001). **(B)** The frequency of peripheral blood (PB, green), tumor (T, red) and tumor-free tissue (TFT, orange) -derived NK cells expressing the indicated surface receptors is shown stratifying CRC patients by tumor stage (I-II and III-IV). Histogram bars represent mean±SD of each surface marker and individual values are indicated by the respective symbols (PB= circles, T=squares, TFT=triangles). Comparisons were performed between different compartments (PB vs T vs TFT) within the same tumor stage through Kruskal-Wallis test followed by Dunn’s test for multiple comparisons, and in the same compartment between stage I-II and III-IV CRC patients by multiple Mann-Whitney test with Holm-Šídák test (stage I-II PB/T n=38, TFT n=20 and stage III-IV PB/T n=30 TFT n=13). Statistical significance calculated by the corresponding test is indicated (*p<0.05; **p<0.01; ***p<0.001; ****p<0.0001).

We first evaluated NK cell frequency in peripheral blood (PB), tumor-tissue (T) and tumor-free tissue (TFT) derived from the CRC patients enrolled in this study (n=80, **Table 1**) by cytofluorimetric analyses. To identify NK cells, we gated on viable CD45^+^Lin^–^(CD3^–^CD19^–^CD14^–^CD33^–^) CD127^–^CD56^+^ lymphoid cells (**Supplementary Figure 1A**) in each compartment. It’s to be noted that most gated cells, both in T and in TFT, expressed the transcription factor Eomes that preferentially marks NK cells rather than ILC1 in tissues (6, 9) (**Supplementary Figure 2**). We found that NK cells (among CD45^+^ lymphoid cells) were present in similar proportions in both T and TFT, although at significantly lower levels compared to PB (**Figure 1A**). Although NK cell frequencies were quite heterogeneous in tissue samples (**Figure 1A**), we were able to analyze NK cells in most CRC patients enrolled (n=68, see also Methods).

### 3.2 NK cells expressing immune checkpoints are enriched in tumor tissues from CRC patients independently from the tumor stage

We performed an in-depth analysis of the surface expression of critical ICs that may impair NK-mediated antitumor response, including HLA-I specific (iKIRs and NKG2A) and non-HLA-I specific inhibitory receptors (PD-1, TIM-3, LAG-3 and TIGIT) by multiparametric flow cytometry. We compared tumor-derived NK cells (T-NK) with those derived from tumor-free tissues (TFT-NK) and peripheral blood (PB-NK), stratifying CRC patients according to their tumor stage (early stages I-II vs advanced stages III-IV). Interestingly, we observed that KIRs, TIM-3 and NKG2A could be detected on a significantly larger fraction of T-NK cells as compared to TFT-NK cells, while PB-NK cells displayed the highest KIRs and TIM-3 frequencies, regardless of the tumor stage (**Figure 1B**). Notably, the immune checkpoint PD-1 was significantly more expressed on T-NK cells as compared to PB-NK cells regardless of the tumor stage, as well as to TFT-NK cells, although reaching statistical significance only in early-stage CRC patients. At variance with KIRs, PD-1, NKG2A and TIM-3, the inhibitory receptor LAG-3 was significantly more expressed on NK cells in both tissue compartments (T and TFT) compared to PB-NK cells, where LAG-3 was almost undetectable (**Figure 1B**). Differently, TIGIT was detected at low frequencies in all compartments with a tendency to higher proportions at late-stage CRC in PB (**Figure 1B**).

Further receptors relevant to NK cell activity and subsets identification such as NKG2C, CD16, and CD57 were also evaluated. NKG2C was expressed at similar frequencies across all three compartments, regardless of the tumor stage analyzed (**Figure 1B**). At variance with PB-, only a minor fraction of both T- and TFT-NK cells expressed CD16. Likewise, CD57, a marker of terminally differentiated cells in PB, was poorly expressed in NK cells from both tissue compartments. Interestingly, TFT-NK cells expressed CD16 at even lower frequencies than T-NK cells. On the contrary, PB-NK cells largely expressed CD57 indicating, along with high ICs frequencies (except for PD-1 and LAG-3) observed in comparison to tissue-derived NK cells (**Figure 1B**), a highly differentiated profile that can be associated to the advanced age of most patients (**Table 1**) (19).

In general, we could not appreciate any significant difference on NK cells derived from early compared to advanced stage tumors.

### 3.3 PD-1^+^ NK cells are clearly enriched in tumor tissues from MSI-CRC patients

**Figure 2:**
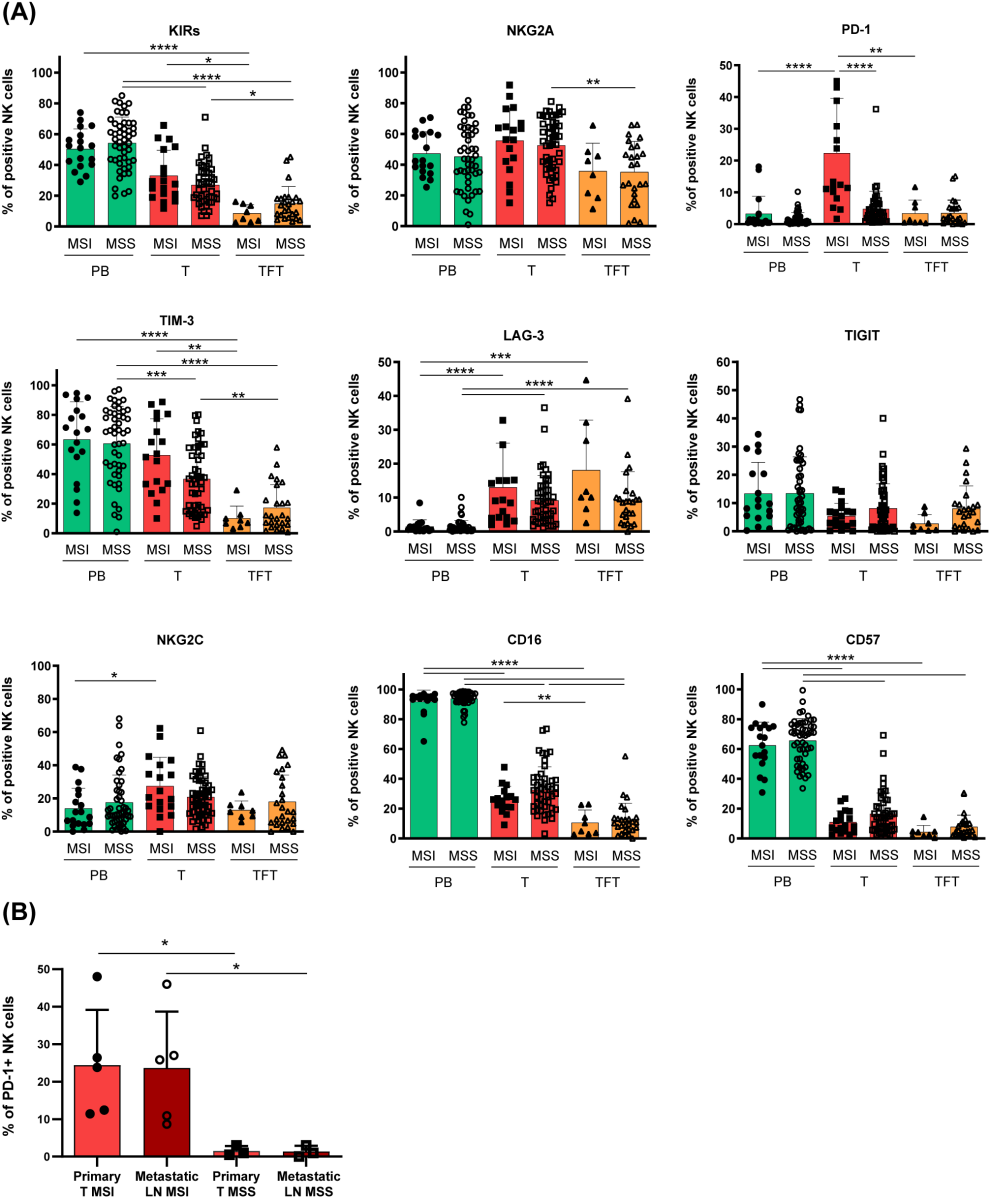
Tumor-associated PD1^+^ NK cells enrichment marks MSI CRC patients. **(A)** The frequency of peripheral blood (PB, green), tumor (T, red) and tumor-free tissue (TFT orange) -derived NK cells expressing the indicated surface receptors is depicted, stratifying CRC patients according to their MMR status, indicated as MSI or MSS (PB/T-MSI n=18; TFT-MSI n=8; PB/T-MSS n=50; TFT-MSS n=25). Histogram bars represent mean±SD of each surface marker and individual values are depicted by the respective symbols (PB=circles, T=squares, TFT=triangles). Expression differences were evaluated by Kruskal-Wallis test followed by Dunn’s test for comparisons between different compartments (PB vs T vs TFT) within the same MMR status group (MSI/MSS) and by multiple Mann-Whitney test with Holm-Šídák test for comparisons between MSI and MSS patients within the same compartment. Statistical significance is indicated (*p<0.05; **p<0.01; ***p<0.001; ****p<0.0001). **(B)** PD-1 expression on primary tumor-NK cells (light red) with respect to NK cells isolated from metastatic lymph nodes (dark red) of MSI (full and empty circles, n=5) and MSS (full and empty squares, n=3) -CRC patients is shown as mean±SD. Statistical significance computed by Mann-Whitney test is indicated (*p<0.05).

Given the efficacy of PD-1/PD-Ls axis blockade (20) in MSI-CRCs patients characterized by abundant PD-1^+^ TILs and following the observation of PD-1^+^ tumor-associated NK cells (**Figure 1B**), we next analyzed PD-1 and the other immune checkpoint expression on T-NK cells, stratifying CRC patients by the MSI/MSS condition. Noticeably, by this analysis we could disclose that the highest fraction of tumor-associated PD-1^+^ NK cells reported in **Figure 1B**, clearly relied on a significant enrichment of PD-1^+^ NK cells in the tumor tissue of MSI CRC as compared to MSS CRC (**Figure 2A**). Importantly, PD-1 expression was significantly higher in T-NK compared to TFT-NK cells only in MSI and not in MSS CRC patients. The surface expression of no other NK cell inhibitory receptor or relevant surface receptor analyzed, significantly correlated with the MSI condition (**Figure 2A**). In line with this observation, all other surface markers examined in T-NK cells from both MSI and MSS CRC patients differed from the corresponding PB- and TFT-NK cells in accordance with the observations reported in **Figure 1B** for the whole cohort divided in early vs advanced stages, without MSI/MSS stratification. We could only observe that in MSI CRC the higher NKG2A expression on T-NK cells than in TFT-NK cells did not reach statistical significance, while NKG2C expression was higher in T-NK than in PB-NK cells, unlike MSS CRC (**Figure 2A**).

Interestingly, in a few metastatic samples available from advanced stage CRC, we could detect a discrete fraction of PD-1^+^ NK cells also in the metastatic lymph nodes from MSI CRC patients (n=5), whereas this subset was virtually absent in both primary and metastatic lesions from MSS CRC patients (n=3) (**Figure 2B**).

### 3.4 Tissue-resident NK cells display a distinct profile in CRC tissues and include tumor- infiltrating PD-1^+^ NK cells in MSI CRC

**Figure 3:**
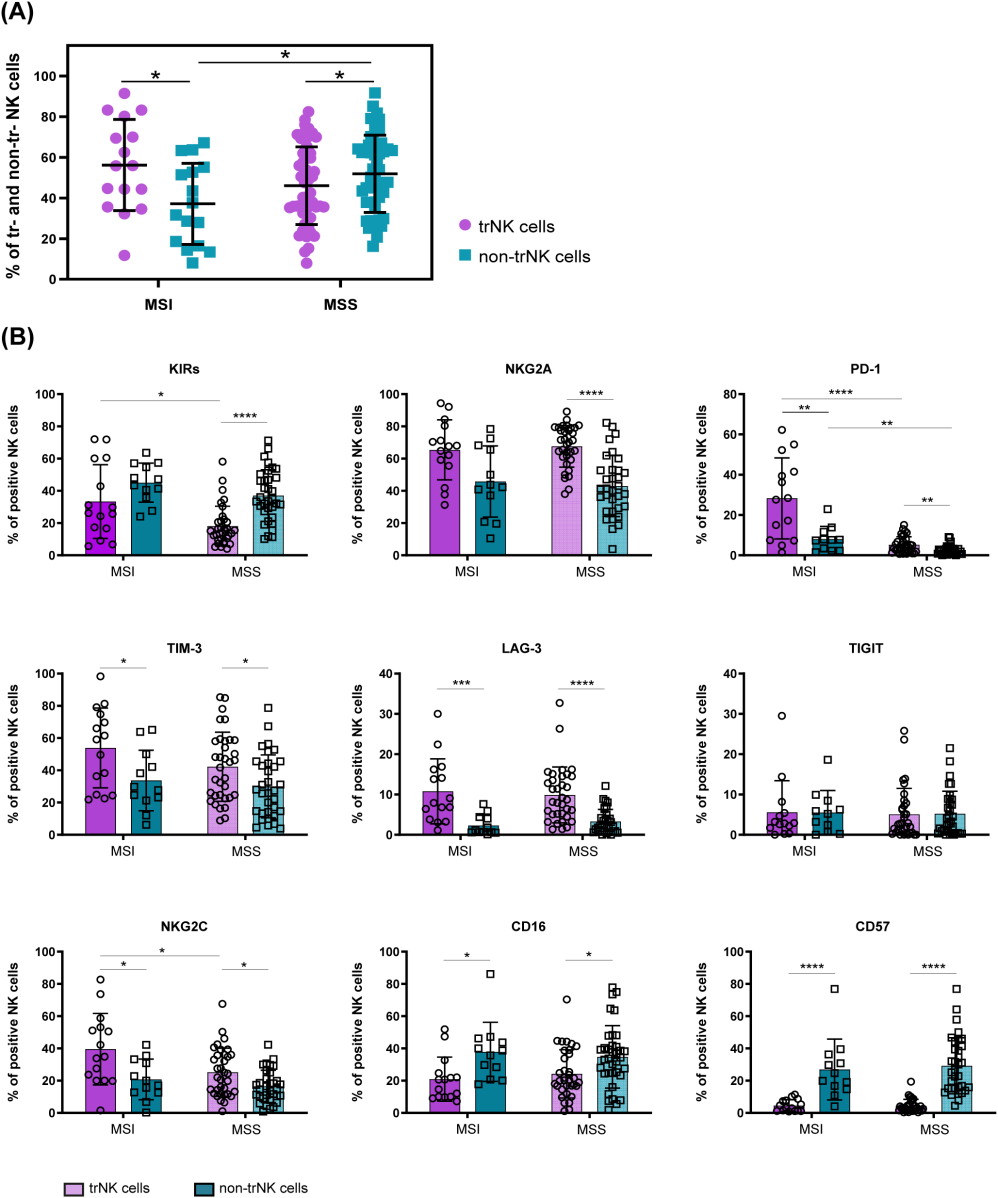
Differential surface signature of tumor-associated trNK cells vs non-trNK cells in MSI vs MSS CRC patients. **(A)** Frequencies of tissue-resident (trNK, purple) and non-tissue resident NK cells (non-trNK, petroleum blue) within the tumor compartment of each MSI (n=16) and MSS (n=52) CRC patient are reported. Vertical bars represent mean±SD. Statistical significance evaluated by Mann-Whitney test is indicated (*p<0.05). **(B)** The frequency of tumor-associated trNK (purple) and non-trNK cells (petroleum blue) in MSI and MSS-CRC patients expressing the indicated cell surface markers is shown (trNK MSI n=15, non-trNK MSI n=12, trNK MSS n=34, non-trNK MSS n=34). Histogram bars represent mean±SD of each surface receptor and single values are indicated by the respective symbols (trNK=circles, non-trNK=squares). Data were analyzed by Mann-Whitney test either between trNK and non-trNK cells within the same MMR status group (MSI/MSS) or between MSI and MSS patients within the same NK cell subset (trNK/non-trNK). Statistical significance is indicated (*p<0.05; **p<0.01; ***p<0.001; ****p<0.0001).

To get further insights into the identity and origin of the heterogeneous ICs^+^ tumor-associated NK cell subsets detected in MSI and MSS CRC, we next characterized T-NK cells based on the expression of the tissue residency markers CD103 and CD49a, known to favor lymphocyte retention in peripheral tissues including the gut (4, 9). In our analyses NK cells expressing one or both these markers (i.e. CD103^+^CD49a^–^, CD103^+^CD49a^+^ and CD103^–^CD49a^+^ NK cells) were considered trNK cells, whereas NK cells lacking both markers (i.e. CD103^–^ CD49a^–^ NK cells) were identified as non-tissue resident NK cells (non-trNK cells) (**Supplementary Figure 1A**). First, we observed that in tumor tissues from MSI CRC patients trNK cells were predominant, at variance with MSS CRCs in which non-trNK cells prevailed (**Figure 3A**). Interestingly, CD69, an additional marker of tissue retention that is also a classic early activation marker, was expressed by both tumor-associated NK-cell subsets, although trNK cells showed significantly higher CD69 frequencies in both patient groups (**Supplementary Figure 3**). We next compared the surface signature of tumor trNK vs non-trNK cells to get insights in their heterogeneity. Notably, we found that PD-1 was expressed mainly by trNK cells in both MSI and MSS CRC patients (**Figure 3B**), although PD-1 was scarcely detectable in MSS as previously observed (**Figure 2A**). TrNK cells were also characterized by higher proportions of LAG-3, TIM-3 and NKG2A, in both MSS and MSI patients (although NKG2A showed only a tendency to higher proportions in MSI patients), suggesting that the expression of these ICs may be induced by the TME (**Figure 3B**). In addition, trNK cells displayed significantly higher proportions of the activating receptor NKG2C as compared to non-trNK cells, in both patient groups, implying a complex role of the TME on tumor-associated NK cell signature, especially in MSI patients where NKG2C^+^ trNK cells are more present compared to MSS.

Interestingly, we also observed that trNK cells expressed KIRs in higher proportions in MSI than in MSS CRC. However, KIR^+^ NK cells were less represented in trNK compared to non-trNK cells for both patient groups, although reaching statistical significance only in MSS CRCs (**Figure 3B**). The different profile shown by trNK cells also involved lower proportions of CD16^+^ and CD57^+^ cells in both MSI and MSS CRC patients (**Figure 3B**).

**Figure 4:**
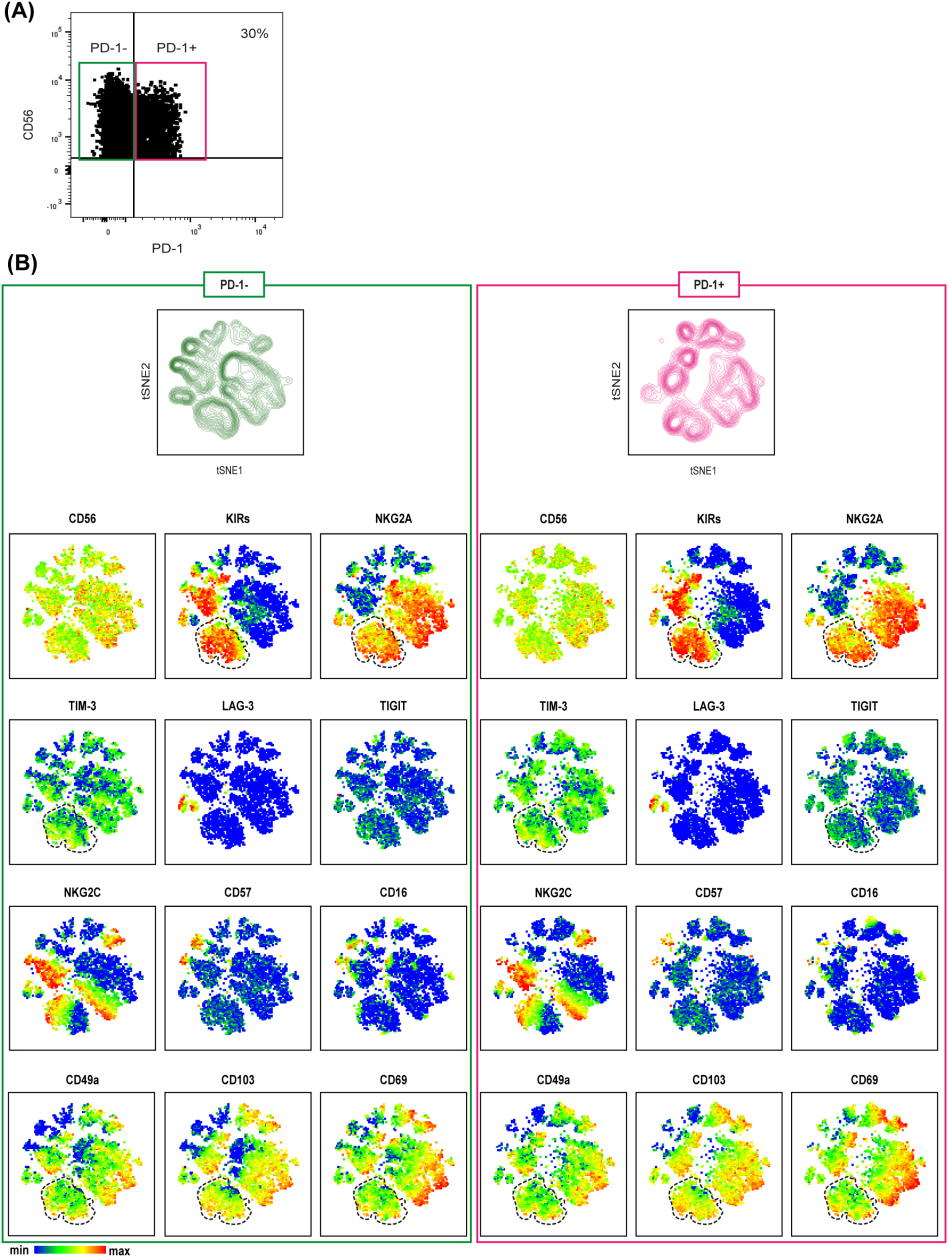
MSI tumor-associated PD-1^+^ NK cells are tissue resident NK cells co-expressing immune checkpoints. **(A)** PD-1 expression on tumor-associated NK cells derived from a representative MSI CRC patient is shown indicating the gating strategy to select PD1^+^ (purple box) and PD-1^─^ (green box) NK cells used for t-SNE analysis. **(B)** t-SNE was generated from an equal number of PD-1^+^ and PD-1^─^ tumor-associated NK cells derived from MSI CRC patients (n=3). The relative spatial distribution of PD1^+^ (purple) and PD-1^─^ (green) NK cells is shown separately in the maps. t-SNE representation of different surface markers on PD-1^+^ (right) and PD-1^─^ (left) tumor-associated NK cells from MSI CRC patients is depicted below. In both PD-1^+^ and PD-1^─^ t-SNE maps, the dotted lines identify a fraction of NK cells co-expressing different ICs, NKG2A, KIRs and TIM-3, also characterized by the expression of the tissue residency markers CD49a, CD103 and CD69.

Finally, to better characterize tumor-associated PD-1^+^ NK cells (**Figures 2A**, **3B**), we performed a t-SNE analysis comparing PD-1^+^ and PD-1^–^ tumor-associated NK cells from MSI CRC patients (**Figure 4**). We could thus confirm that PD-1^+^ NK cells largely expressed tissue residency markers (see also **Supplementary Figure 4A**), including CD69, and more importantly we observed that a discrete fraction of PD-1^+^ NK cells could coexpress additional ICs, such as KIRs, NKG2A and TIM-3 (highlighted in **Figure 4**). In addition, small proportions of PD-1^+^ NK cells coexpressed only KIRs lacking NKG2A, while larger fractions coexpressed NKG2A lacking KIRs (**Figure 4**, **Supplementary Figure 4B**). Overall, PD-1^+^ and PD-1^–^ NK cells did not display statistically significant differences (**Supplementary Figure 4C**). Indeed, we observed that NKG2A, KIRs and TIM-3 were coexpressed also on a large fraction of tumor-associated PD-1^–^ NK cells (**Figure 4**).

### 3.5 Tumor-associated PD-1^+^ tr-NK cells from MSI CRCs are functionally equipped

Since TME can give rise to an “exhausted” NK cell phenotype (21), characterized by both increased ICs expression (including PD-1), and decreased activating NK receptor expression, along with lower functional capabilities, we assessed the surface expression of activating NK receptors and the content of cytotoxic molecules, first comparing PB and tumor-associated trNK cells from MSI and MSS CRC patients.

As shown in **Supplementary Figure 5**, in both MSI and MSS CRCs the activating receptors NKp46 and NKp30 were comparable on PB- and T-NK cells, at variance with NKp44, that was negligible on PB-NK cells and expressed by a small fraction of T-NK cells. In line with previous data (22), we found that the frequency of DNAM-1^+^ NK cells was decreased in T- compared to PB-NK cells in both MSI and MSS patients, while NKG2D^+^ NK cells were lower only on T-NK cells from MSI CRC. Also a lower content in granzyme B and perforin was detected on T-NK cells with respect to PB-NK cells from both patient groups. We also compared PB-NK cells from healthy donors (HD) with patients’ PB-NK cells and observed only small differences in terms of NKp30 levels, a tendency to lower DNAM-1^+^ NK cells proportions in both patient groups, while NKp46, granzyme B and perforin were similarly expressed at variance with previous observations (23).

**Figure 5:**
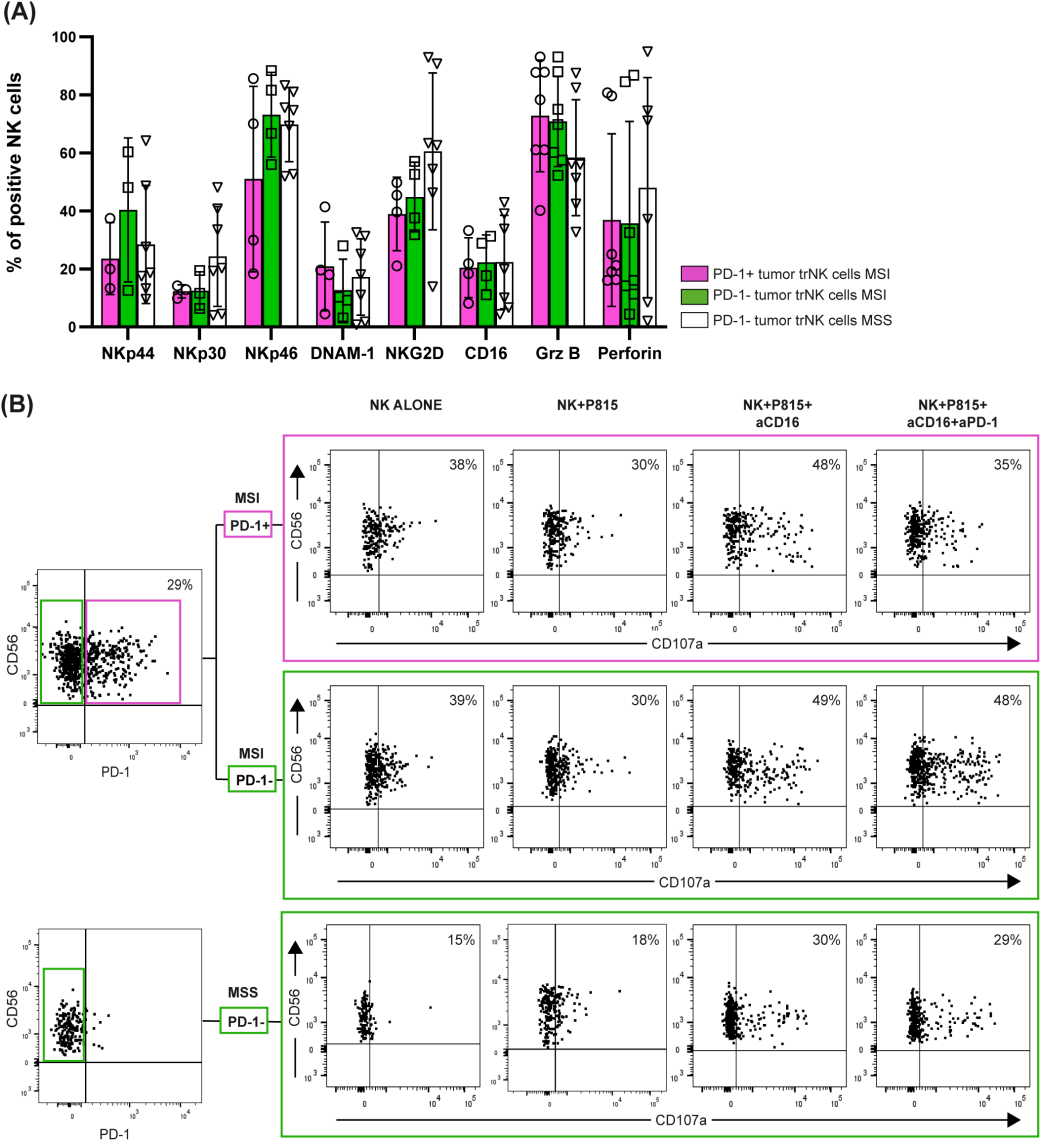
PD-1^+/─^ tumor-associated trNK cells from MSI/MSS CRC patients express activating receptors and display cytotoxic potential. **(A)** Surface expression of NK activating receptors (actR) (NKp44, NKp30, NKp46, DNAM-1, NKG2D, CD16) and intracellular expression of granzyme B (Grz B) and perforin (intra) were evaluated in tumor-associated PD-1^+^ (purple bars, circles) and PD-1^─^ (green bars, squares) trNK cells from MSI CRC patients, in comparison to PD-1^─^ trNK cells from MSS CRC patients (white bars, triangles) (actR MSI n=4, actR MSS n=7, intra MSI n=7, intra MSS n=7). Histogram bars represent mean±SD and single sample values are indicated by the corresponding symbol. No statistical significance was found. **(B)** A degranulation assay of tumor-associated PD-1^+^ (purple box) and PD-1^─^ (upper green box) trNK cells from a representative MSI CRC patient and of PD-1^─^ trNK cells (lower green box) from a representative MSS CRC patient is shown. Patients’ NK cells were cultured alone (negative control) or co-cultured with the FcγR^+^ P815 murine cell line for 3 hours (E:T ratio 1:1), in presence or absence of a mAb targeting CD16 (aCD16) or of a combination of aCD16 and a mAb targeting PD-1 (aPD-1). Percentages of degranulating CD107a^+^ NK cells are reported in the upper right quadrant for each condition. The CD107a marker was positioned according to the respective isotype control (not shown).

Next, by focusing on tumor-associated PD-1^+^ tr-NK cells from MSI CRC patients, we found that the expression of activating receptors (NKp46, NKp30, NKp44, DNAM-1, NKG2D and CD16) was similar or only slightly lower on tumor-associated PD-1^+^ tr-NK cells from MSI CRCs compared to tumor-associated PD-1^–^ tr-NK cells from both MSI and MSS CRCs. We also observed similar proportions of tr-NK cells from MSS and MSI patients expressing granzyme B and perforin in all PD-1^+/-^ subsets (**Figure 5A**, **Supplementary Figure 5B**).

We further assessed PD-1 functionality on tumor-associated PD-1^+^ tr-NK cells from a representative MSI CRC patient from whom we were able to obtain a sufficient number of NK cells after tissue dissociation. By a degranulation assay performed against the FcγR^+^ P815 cell line, we could prove that PD-1^+^ tr-NK cells responded to mAb-mediated CD16 triggering and that the simultaneous triggering of PD-1 decreased CD16-induced stimulation (**Figure 5B**), thus suggesting that tumor-associated PD-1^+^ tr-NK cells expressed a functional PD-1 inhibitory receptor. As expected, PD-1 triggering could not inhibit degranulation on PD-1^–^ tr-NK cells from either the same MSI patient or a representative MSS patient (**Figure 5B**).

Notably, in this representative patient, PD-1 triggering decreased PD-1^+^ tr-NK cells degranulation (**Figure 5B**) despite the modest PD-1 expression level on trNK cells, compared to the high expression level that characterized PD-1^+^ tumor-associated T cells from the same MSI CRC patients (**Supplementary Figure 6**).

### 3.6 Enrichment of PDL1–2 on tumor cells derived from patients with MSI CRC

In light of the finding of CRC-associated tr-NK and non-tr-NK cells expressing PD-1 and/or KIRs and NKG2A, we analyzed the surface expression of their respective ligands, PD-Ls and HLA-I molecules, on tumor cells derived from CRC samples (identified as CD45^–^ EpCAM^+^ CD90^–^ cells, **Supplementary Figure 1B**), to evaluate whether those critical ICs might play a role in regulating NK cell-mediated antitumor responses against CRC.

**Figure 6:**
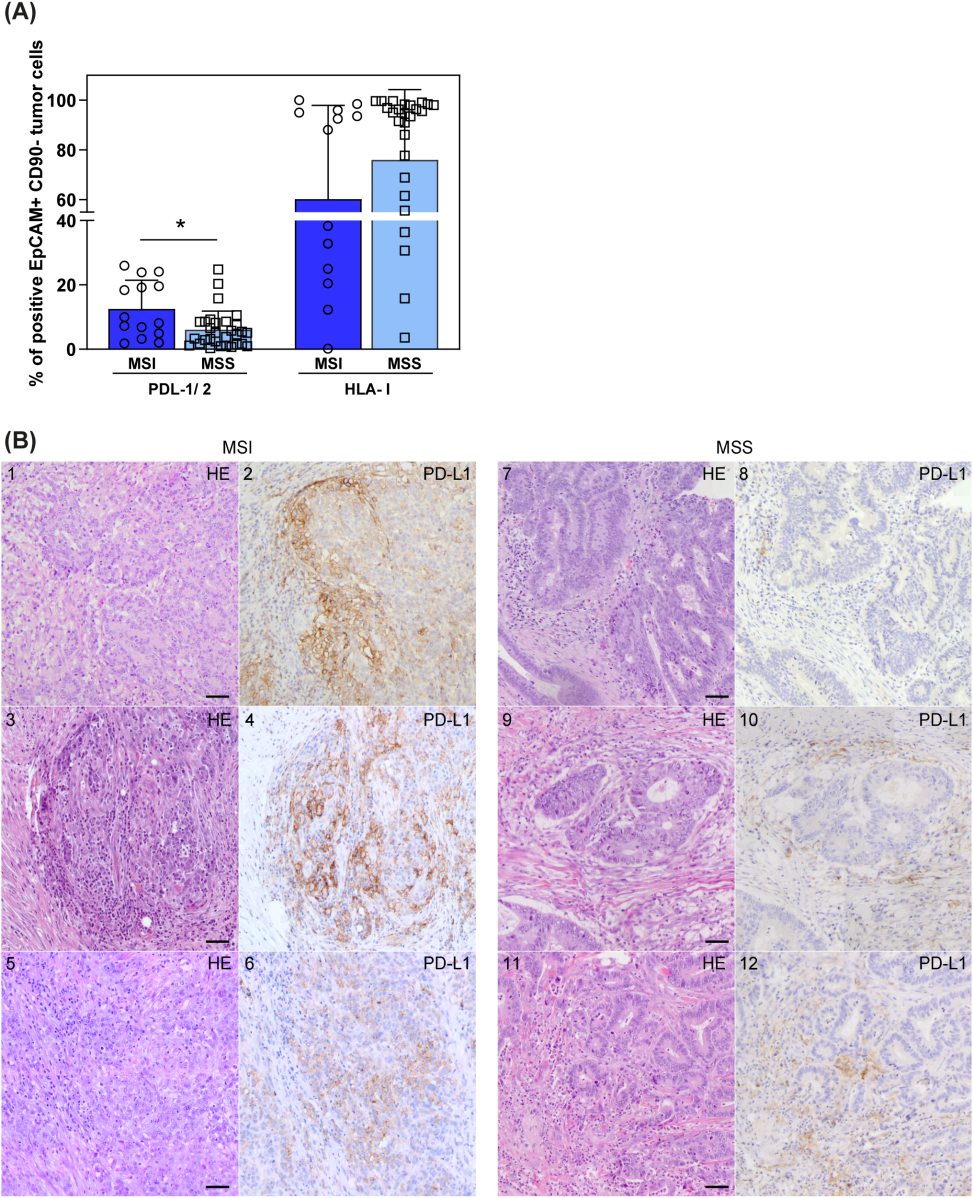
PD-L1/2 and HLA-I expression on tumor cells in MSI vs MSS CRC patients. **(A)** Surface expression of PDL-1/2 and HLA-I on EpCAM^+^CD90^─^ tumor cells, derived from CRC patients stratified by MMR status (MSI n=14, blue, circles; MSS n=30, light blue, squares), was investigated by cytofluorimetric analyses. Histogram bars represent mean±SD and single values are depicted. Statistical significance calculated with Mann-Whitney test is indicated (* p<0.05). **(B)** Tumor tissue sections from six representative CRC patients, stained with anti-PD-L1 by IHC, are depicted. 1–6 show sections from MSI CRC patients, 7–12 show sections from MSS CRC patients (1, 3, 5, 7, 9, 11 panels show HE staining; 2, 4, 6, 8, 10, 12 show anti-PD-L1 IHC staining). Scale bars are 50 µm. Magnification: 20X HE, hematoxylin-eosin staining; IHC, immunohistochemistry.

Interestingly, our cytofluorimetric analyses displayed a significantly higher expression of PD-Ls on tumor cells derived from MSI CRCs compared to MSS CRCs. In contrast, HLA-I expression tended to be lower on MSI CRCs compared to MSS CRCs, although not significantly (**Figure 6A**). In line with cytofluorimetric data, increased PD-L1 expression on tumor cells from MSI CRC (**Figures 6B**2, 4, 6) compared to MSS CRC (**Figures 6B**8, 10, 12) was also observed by IHC analysis in agreement with previous studies (24, 25). In particular, in MSI CRCs, PD-L1 was detected at high levels on tumor cells and to a lesser extent on leukocytes located mainly in the peritumoral area (**Supplementary Figures 7**).

## 4 Discussion

High numbers of tumor-associated CD8^+^ and CD4^+^ T cells, with Th1 profile, and of PB- and tumor-associated NK cells have been correlated with better prognosis in CRC (1, 14, 26). It is now increasingly clear that both the complex interplay between tumor-associated immune cells and their profile in the TME can influence the outcome of immunotherapy also in regard to distinct TIL differentiation programs, characterized by residency and/or exhaustion markers (e.g., T cells upregulate inhibitory ICs such as PD-1) and loss of anti-tumor function (14, 27, 28). However, these programs, well-studied in CD8^+^ T cells (27, 28), remain less understood in tumor-associated NK cells, including CRC, highlighting the need for detailed investigations into tumor-associated NK cell features that will help design novel combination treatments incorporating NK-based immunotherapies.

In the present study, through in-depth cytofluorimetric analysis of the expression of different ICs that critically regulate NK cell function, we observed a significant enrichment of tumor-associated NK cells expressing certain ICs (i.e., KIR, NKG2A, and TIM-3) compared to NK cells from tumor-free tissue, along with an increase in PD-1^+^ NK cells in tumor tissue, compared to both PB and tumor-free tissue. In line with our observations, PD-1^+^ NK cells had been already detected in patients with other solid tumors, such as ovarian cancer, peritoneal carcinomatosis and Kaposi sarcoma (8, 29). Remarkably, our data clearly showed that tumor-associated PD-1^+^ NK cells were significantly enriched in MSI CRC than in MSS CRCs, both in primary and metastatic lesions. Notably, patients with MSI CRC and especially those with metastatic CRC (mCRCs), have shown a great benefit following immunotherapeutic treatments based on the blockade of the PD-1/PD-Ls interaction with Pembrolizumab (anti-PD-1 mAb), demonstrating superior progression-free survival (PFS) compared to chemotherapy in patients with newly-diagnosed dMMR mCRC (20, 30). It is therefore conceivable that the efficacy of Pembrolizumab treatment in these patients may rely not only on the enhanced function of PD-1^+^ T cells, known to infiltrate MSI CRC (31, 32), but also on a superior antitumor potential exerted by PD-1^+^ NK cells following mAb-mediated blockade of the PD-1/PD-Ls interaction.

In our analyses we found that in MSI CRCs a negligible fraction of these tumor-associated PD-1^+^ NK cells expressed CD57 (classic marker of terminally differentiated NK cells in PB), while the majority of them expressed the tissue residency markers CD103 and/or CD49a (trNK cells). These features suggest that tumor-associated PD-1^+^ trNK cells may represent NK cells recruited from circulation, retained in tumor tissue, and reconfigured by TME signals, whereas tumor-associated PD-1^–^ NK cells that do not express tissue-residency markers (and which include almost all CD57^+^ NK cells) may represent recently recruited NK cells not yet exposed to the TME. In line with this hypothesis, circulating PB-NK cells have been shown to acquire the tissue-residency markers CD49a and CD103 *in vitro* after 6 days of culture in the presence of IL-15 and tumor cell lines (33). Notably, trNK cells were detectable in significant proportions also in MSS CRC, although characterized by lower KIR and negligible PD-1 expression compared to trNK cells from MSI CRC. In addition, lung cancer-infiltrating NK cells expressing CD49a and CD103 have been observed at a higher percentage in the tumor center than at the tumor margin and scarcely in the peritumoral region, again suggesting a role for the TME on NK cell signature (34). A detailed analysis of chemokine receptor expression in tumor-infiltrating NK cells will be mandatory to fully disclose the origin and distribution of the various tumor NK cell subsets identified here. In this context, although we analyzed bona fide NK cells by gating on Lin^–^CD56^+^ lymphocytes lacking CD127, a conventional marker for helper ILC (35–37), and also expressing largely Eomes, we cannot exclude that a minor fraction of tissue resident CD56^+^ cells may represent intraepithelial ILC1 (ieILC1), a subset of mucosal ILC lacking CD127 and sharing several traits with NK cells (31, 37). Indeed, we observed in some tumor samples a minor subset of CD56^+^Lin^–^CD127^–^ cells expressing both the tissue residency markers CD103/CD49a and NKp44 (**Figure 5A**), thus partially resembling tonsil ieILC1 (37) or possibly representing an intermediate NK/ILC1 population that deserves further analyses to be precisely identified.

Interestingly, a recent manuscript showed that MSI CRC patients with higher frequencies of tumor-infiltrating PD-1^+^ NK and NKT cells had significantly longer disease-free survival (DFS) and suggested an anti-inflammatory role for these cells (38). However, tumor-associated NK cells, identified as CD3^–^CD56^+^ cells, were barely characterized phenotypically in that study and the possible anti-tumor role of tumor-infiltrating PD-1^+^ NK cells was not considered.

**Figure 7:**
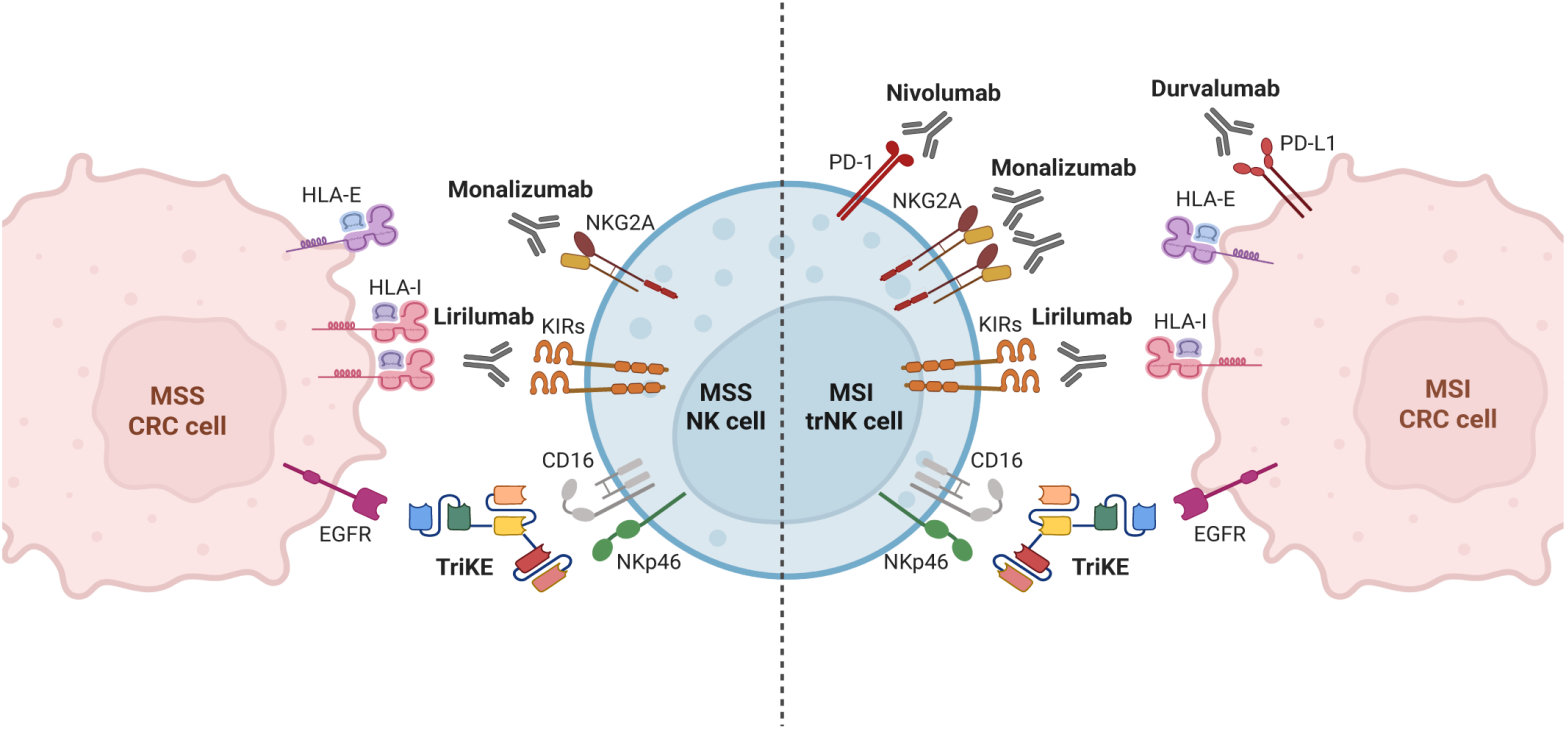
Strategies to enhance tumor-associated NK cell function against MSI/MSS colorectal cancer. Strategies aimed at reducing the suppressive effect exerted by interactions of immune checkpoints expressed on NK cells with their ligands on tumor cells or at triggering the antitumor potential of NK cells could be combined differently depending on the characteristics of NK cells in different CRC type, to achieve the best immunotherapeutic effect. Based on our observations, the antitumor efficacy of tumor-associated NK cells can be potentiated through: i) IC blockade via anti-NKG2A(Monalizumab), anti-KIRs (Lirilumab) and anti-PD-1/PD-L1 (Nivolumab/Durvalumab) mAbs in MSI CRC; ii) IC blockade via anti-NKG2A (Monalizumab) and anti-KIRs (Lirilumab) mAbs in MSS CRC; iii) ADCC triggering strategies via trifunctional NK cell engagers (TriKE) targeting CD16 and NKp46 on NK cells and a tumor-associated antigen (such as EGFR) on tumor cells, regardless MSI/MSS status. Created in BioRender. Della chiesa, M. (2025) https://BioRender.com/yxsy628.

In this context, we found that tumor-associated PD-1^+^ trNK cells from MSI CRCs, as well as PD-1^–^ trNK cells from MSI and MSS CRC, were characterized by adequate amounts of activating NK receptors and a moderate but consistent content of perforin/granzyme B, to exert anti-tumor activities. However, both DNAM-1 expression and cytotoxic molecule content were lower than in PB-NK cells, in line with previous data and with an immunosuppressive effect of the TME (22, 23). Of note, in our cohort we did not observe the sharp dysregulation in surface expression of NCRs and NKG2D that had been previously described in CRC PB-NK compared to HD PB-NK (22, 23) but only a slight decrease in the expression of NKp30 and DNAM-1. Furthermore, we observed a substantial level of NKp46 expression on PB and tumor-associated NK cells in both MSS and MSI CRC (**Supplementary Figure 4A**). Taken together, these findings suggest that both PB and CRC-infiltrating NK cells are sufficiently equipped to design immunotherapies aimed at enhancing and exploiting their anti-tumor activity. In this context, the main strategies to boost NK-cell function against CRC could rely on the use of mAbs or BiKEs targeting CRC tumor antigens (e.g., EGFR, CEA, HER2, MUC-1 and LGR5) that can directly induce tumor cell death but also simultaneously trigger NK cell cytotoxicity via ADCC (39). However, due to low expression of CD16 but significant expression of NKp46 on tumor-associated NK cells (**Figures 1B**, **5A**), the use of novel trifunctional NK cell engagers simultaneously triggering NKp46 and CD16 on NK cells, and a tumor antigen on CRC cells (e.g. EGFR), could represent a more suitable approach to effectively control tumor growth (39, 40) (summarized in **Figure 7**). Another promising strategy to enhance NK cell activity against CRC is the adoptive transfer of NK cells engineered with chimeric antigen receptors (CARs). CAR-NK cells have been designed to target specific cancer antigens, which are highly expressed on most CRC, such as EpCAM, CEA, NKG2D ligands (through an NKG2D-CAR construct), and MUC-1 (39, 41), or to target antigens expressed only on a fraction of CRC patients, such as HER-2 (42) or Mesotelin (43). On the other hand, the use of CAR-NK for the treatment of solid tumors such as CRC is challenging due to the poor homing capabilities and reduced anti-tumor activity of CAR-NKs within the immunosuppressive environment of the tumor site (41). However, this approach could be combined with the use of NK cell-engagers and/or other strategies to enhance the antitumor function of NK cells, such as mAb-mediated IC blockade. As mentioned, this treatment could unleash the potential of the tumor-associated IC^+^ NK cells (depicted in **Figure 7**). Regarding IC blockade efficiency, it must be pointed out that although Pembrolizumab-mediated blockade of PD-1/PD-Ls interaction has demonstrated efficacy in patients with mCRCs (20, 30), MSI status alone may not be sufficiently predictive of response to PD-1/PD-Ls inhibitors. Indeed, not all patients with MSI mCRCs respond to immunotherapy and some patients show resistance to ICIs (20, 30), therefore suggesting that other parameters may interfere with the efficacy of PD-1/PD-Ls blockade in this subgroup of patients. On the other hand, PD-1/PD-Ls blockade can provide benefits also in a fraction of MSS/MMR proficient CRC patients, characterized by high TMB and increased immune cells infiltration, similarly to MSI tumors (44). Thus, it is essential to define additional criteria to design the most suitable therapy. In this context, our data show that a large proportion of tumor-associated PD-1^+^ tr-NK cells coexpressed additional ICs, including those that typically inhibit NK cell function following HLA-I recognition, that is the inhibitory receptors KIRs and NKG2A. Thus, the combined blockade of PD-1, KIRs and NKG2A could further improve the NK cell-mediated antitumor response, compared to PD-1/PDLs blockade alone. KIRs and NKG2A were also largely expressed on tumor-associated PD-1^–^ NK cells in both MSI and MSS CRCs, regardless of whether they expressed tissue residence markers or not. Therefore, the use of specific ICIs that block NKG2A and/or KIRs could enlarge the NK-cell subset capable of fighting MSI CRCs (PD-1^+^ KIR^–^ NKG2A^–^ plus PD-1^+^ KIR^+^ and/or NKG2A^+^ plus PD-1^–^ KIR^+^ and/or NKG2A^+^ NK cells) and could be also effective against MSS CRCs where tumor-associated PD-1^+^ NK cells are scarce. Two clinical trials evaluating the combination of an anti-NKG2A antibody with anti PD-1/PD-L1/CTLA-4 for the treatment of solid tumors, including MSS CRC, are currently ongoing. The first one is a phase 1–2 trial exploring the combination of a standard treatment (chemotherapy +/- biological agents) plus Durvalumab (anti-PD-L1 mAb) and Monalizumab (anti-NKG2A mAb) in patients with selected advanced solid tumors (including CRC) who have not received prior immunotherapy. Interestingly, some preliminary data have shown a good tolerability profile of the combination treatment, activation of immune cells including CD56^+^CD16^–^ PB-NK cells and a trend towards higher numbers of tumor-associated NKp46^+^ NK cells together with significantly higher numbers of tumor-associated CD8^+^ T cells (45) (NCT02671435). The second trial, not yet recruiting, will explore the efficacy of the combination of Monalizumab and MEDI5752 (a bispecific antibody that targets both PD-1 and CTLA-4) in metastatic dMMR/MSI solid tumors, including CRC (NCT06152523). Notably, a phase I trial (NCT05162755) that combines NKG2A and PD-1 blockade with anti-EGFR is currently active for patients with metastatic gastric tumor and CRC. In addition, Lirilumab, an anti-KIR blocking antibody that targets the inhibitory KIR2DL1, -2 and -3 receptors (and the activating receptors KIR2DS1 and -2) is also available and has shown limited side effects (46), but unfortunately also limited efficacy in the treatment of patients with hematologic malignancies (such as acute myeloid leukemia and myeloma) and some solid tumors. However, Lirilumab in combination with Nivolumab, an anti-PD-1 mAb, showed benefits in a phase II clinical trial for recurrent, resectable squamous cell carcinoma of head and neck (47). Further immunotherapeutic approaches aimed at increasing the anti-tumor potential of NK and T cells should also combine IC blockade and/or triggering mAbs, while simultaneously mitigating the suppressive effects of the TME (39). In this context, our analyses in CRC has clearly showed that tumor-associated NK cells display a higher expression of several ICs (such as KIRs, TIM-3, and NKG2A in all CRC and PD-1 only in MSI CRC) compared to TFT-NK cells, suggesting a modulatory effect of the TME on the NK cell landscape, in line with a role proposed for miRNAs with immune-modulating effects on KIRs expression (48) or for glucocorticoids combined with IL-12, IL-15 and IL-18 inducing PD-1 surface expression (49). However, the exact mechanisms underlying the preferential induction of PD-1 expression on trNK cells in MSI rather than MSS CRC are not yet clearly known. Moreover, hypoxia, soluble factors (such as HIF-α, TGF-β, IL-10, and IL-27) (50) and cancer-derived circRNA present in the TME have been shown to induce ICs expression on T cells (51).

In keeping with the critical role of the PD-1/PD-L1 axis, we also observed a moderate but significant enrichment of PD-L1/2^+^ tumor cells in MSI vs MSS CRCs by cytofluorimetric analysis, also confirmed by IHC analysis, in line with previous studies (21, 25). In this regard, it is important to consider that the level of PD-L1 expression on cancer cells as a predictive biomarker for the efficacy of PD-1/PD-Ls blockade has been proved by IHC in many trials, across different types of solid tumors, in particular melanoma and Non-Small Cell Lung Cancer (NSCLC) (52, 53). However, not all the patients positive for PD-L1 by IHC analysis respond to ICIs, again suggesting the need for additional criteria to select the most suitable immunotherapeutic approach (54).

In addition, to fully exploit the antitumor function of both NK and T cells, it will be crucial to evaluate the impact of the crosstalk occurring between different lymphocyte populations, including iNKT cells, and other immune cells that populate the TME in CRC such as dendritic cells (DC). Indeed, iNKT-DC as well as NK-DC reciprocal interactions could be exploited to design novel alternative therapies to counteract CRC, as recently suggested (55).

Overall, our results highlight that a thorough assessment of tumor-associated immune cell characteristics (including NK cells), coupled with the evaluation of parameters such as PD-L and HLA-I expression, as well as the MSI/MSS status, could improve therapeutic outcomes. In particular, the best therapeutic approach could be achieved by combining cell engagers with NKG2A/KIRs blockade in MSS CRC or with both PD-1/PD-L1 and NKG2A/KIRs blockade in MSI CRC. Therefore, tailoring immunotherapies based on CRC-type-related NK cell characteristics (e.g., IC co-expression) could overcome currently used therapies, by better exploiting the antitumor activity of circulating NK cells and, more importantly, TME-resident NK cell subtypes.

However, this study has some limitations due to the sample size and the limited number of tumor-associated NK cells available to perform functional analyses. Indeed, obtaining sufficient numbers of tumor-associated NK cells from small tumor fragments of MSI CRC is a significant challenge, even considering the low frequency of MSI CRCs. In addition, future studies are needed to assess the suitability of our findings in a more diverse patient population, in order to evaluate the impact of race and ethnicity in the design of more targeted immunotherapies. Nevertheless, the analyzed cohort was sufficient to observe statistical significance for many relevant outcomes, and we believe that the obtained results may provide valuable insights into the phenotype of NK cell within the TME of CRC, useful to guide future translational clinical studies.

## Data Availability

The original contributions presented in the study are included in the article/**Supplementary Material**. Further inquiries can be directed to the corresponding authors.
