# Supplementary material for: Colorectal cancer-infiltrating NK cell landscape analysis unravels tissue-resident PD-1+ NK cells in microsatellite instability tumors
Source: Front Immunol. 2025 Jun 18;16:1578444. doi: 10.3389/fimmu.2025.1578444 (PMC12213648; doi:10.3389/fimmu.2025.1578444)
Supplement: Supplementary file 1 [file DataSheet1.pdf]

Supplementary Material

Colorectal cancer-infiltrating NK cell landscape analysis unravels tissue-resident PD-1$^+$ NK cells in microsatellite instability tumors

Valentina Obino[1^], Chiara Giordano[1^], Simona Carlomagno[2], Chiara Setti[1], Marco Greppi[1], Matteo Bozzo[3], Silvia Pesce[1,4], Elisa Ferretti[1,5], Simona Candiani[3,4], Letizia Muccio[1], Enrico Ciferri[6], Tania Buttiron Webber[7], Agnese Solari[1], Fulvia Ortolani[2], Laura Paleari[8], Matteo Clavarezza[7], Andrea Barberis[6], Marco Filauro[6], Nicoletta Provinciali[1,7], Mariangela Rutigliani[9], Emanuela Marcenaro[1,4*], Andrea De Censi[7,10], Mariella Della Chiesa[1,4*#], Simona Sivori[1,4*#]

1 Supplementary methods

1.1 Flow cytometry and monoclonal antibodies

Phenotypic analyses of surface and intracellular markers on PBMCs and cell suspensions of tumor and tumor-free tissues were performed by multiparametric flow cytometry on a 18-colors LSR Fortessa flow cytometer (BD Biosciences, San Jose, CA, USA). All monoclonal antibodies (mAbs) used in this study are reported below in Supplementary Table 1 (Table S1).

Briefly, cells were incubated for 30 minutes at 4°C with fluorescently labeled mAbs diluted in Brilliant Stain Buffer (BD Biosciences) to prevent unspecific cross-reactions, washed and subsequently labeled with BD Horizon™ Fixable Viability Stain 620 (BD Biosciences) according to the manufacturer's instructions to exclude dead cells. For tumor cells analyses, the Green Fluorescent Nucleic Acid Stain SYTO™ 16 (ThermoFisher Scientific, Waltham, Massachusetts, USA) was added according to the manufacturer's instructions to select nucleated cells among CD45$^-$ cells. For intracellular staining (i.e., granzyme B and perforin), cell fixation, permeabilization and washing were performed using the FoxP3 Staining Buffer Set (Miltenyi Biotec) according to the manufacturer's instructions.

Flow cytometric data were analyzed with FlowJo™ v10.9 software (BD Biosciences). NK cells were identified by gating on viable CD45$^+$Lin$^-$ (CD3$^-$ CD19$^-$ CD14$^-$ CD33$^-$), CD127$^-$, CD56$^+$CD16$^{+/-}$ lymphoid cells (Suppl. Figure 1A), tissue-resident NK cells were identified by gating subsequently on CD103$^+$ and/or CD49a$^+$ NK cells (Suppl. Figure 1A). For intracellular molecules and activating receptors staining, NK cells were identified gating on CD45$^+$, viable, CD3$^-$ Lineage$^-$ (CD19, CD14, CD33, CD127 in the same fluorescence channel) CD56$^+$ CD16$^{+/-}$ lymphoid cells, while T cells were selected as CD45$^+$, viable, Lineage$^-$ (CD19, CD14, CD33)

$CD127^{+/-}$ $CD3^{+}$ cells (Suppl. Figure 1a box). Tumor cells were identified by gating on viable nucleated ($SYTO16^{+}$) $CD45^{-}$ $EpCAM^{+}$ $CD90^{-}$ cells (Suppl. Figure 1B).

During data analysis, a cut-off of 200 cells in the selected gate (NK or tumor cells gates, Suppl. Fig. 1A, B) was applied for each analysis performed, therefore samples not reaching the minimum cell number were excluded. Sample numbers considered in each evaluation are specifically indicated in the corresponding figure legends.

For t-distributed stochastic neighbor-embedding (t-SNE) representation of $PD\text{-}1^{+}$ and $PD\text{-}1^{-}$ tumor-associated NK cells, t-SNE algorithm available in FlowJo™ v10.9 software (BD Biosciences) was used. Equal numbers of $PD\text{-}1^{+}$ and $PD\text{-}1^{-}$ NK cells (1000 each) from 3 different MSI-CRC patients were concatenated through the "Concatenate" function of FlowJo™ software and t-SNE was computed setting a perplexity value of 30, a vantage point tree KNN-algorithm and the Barnes-Hut gradient algorithm.

1.2 CD107a degranulation assay

For functional evaluation of tumor-associated NK cells, CRC-derived single cell suspensions were thawed and underwent removal of cell debris with Debris Removal Solution (Miltenyi Biotec). The percentage of lymphocytes and NK cells within the tumor-derived cell suspensions was evaluated before proceeding further, verifying the almost total absence of tumor cells in the sample after defrosting. In parallel, PBMCs of the corresponding CRC patients (PB) and PBMCs from HDs were thawed and similarly treated in the following steps. Subsequently, patients and HD cells were cultured in complete medium with rhIL-15 1 ng/ml (Peprotech, London, UK), overnight at 37°C and then recovered, washed and counted. PB, HD and tumor NK cells were incubated for 3 hours with anti-CD107a-PE mAb (BD Biosciences) in complete culture medium, alone, as negative control, or with the $Fc\gamma R^{+}$ P815 mastocytoma cell line (E:T ratio 1:1), in presence or absence of an anti-CD16 mAb (c127, IgG1) or with a combination of anti-CD16 and anti-PD-1 mAbs (PD-1.3.1.3 clone, IgG2b, Miltenyi Biotec) (Table S1). Thereafter, NK cells were stained with the appropriate fluorescent-labeled mAbs (Table S1) as previously described and analyzed by flow cytometry.

1.3 Immunohistochemistry and MSI/MSS state assessment

Immunohistochemical stainings were performed on 2 μm thick FFPE sections with the Ventana BenchMark ULTRA automated IHC staining system (Roche Diagnostics, Basel, Switzerland) using the ultraView Universal DAB detection Kit (Roche Diagnostics, Basel, Switzerland) and the following antibodies from Ventana (Roche Diagnostics, Basel, Switzerland): CD45 (clone 2B11 &PD7/26), PD-L1 (clone SP263), MSH6 (clone SP93), MLH1 (clone M1), MSH2 (clone G219-

1129), and PMS2 (clone A16-4). Heat-induced epitope retrieval was performed using the Ventana CC1 buffer (Roche Diagnostics, Basel, Switzerland). Stainings were performed according to the manufacturer's recommendations. Slides were digitalized at 10X magnification using an Olympus BX60 and the Microvisioneer Manual WSI software (Microvisioneer, Wasserburg am Inn, Germany).

Microsatellite status was evaluated by sequential immunohistochemistry of the mismatch repair (MMR) proteins MLH1, MSH2, PMS2, and MSH6. MSI/MMR deficiency was defined as the loss of one or more MMR proteins, while MSS/MMR proficiency was defined as the expression of all four MMR proteins.

2 Supplementary figures and tables

2.1 Supplementary figures

Supplementary figure 1

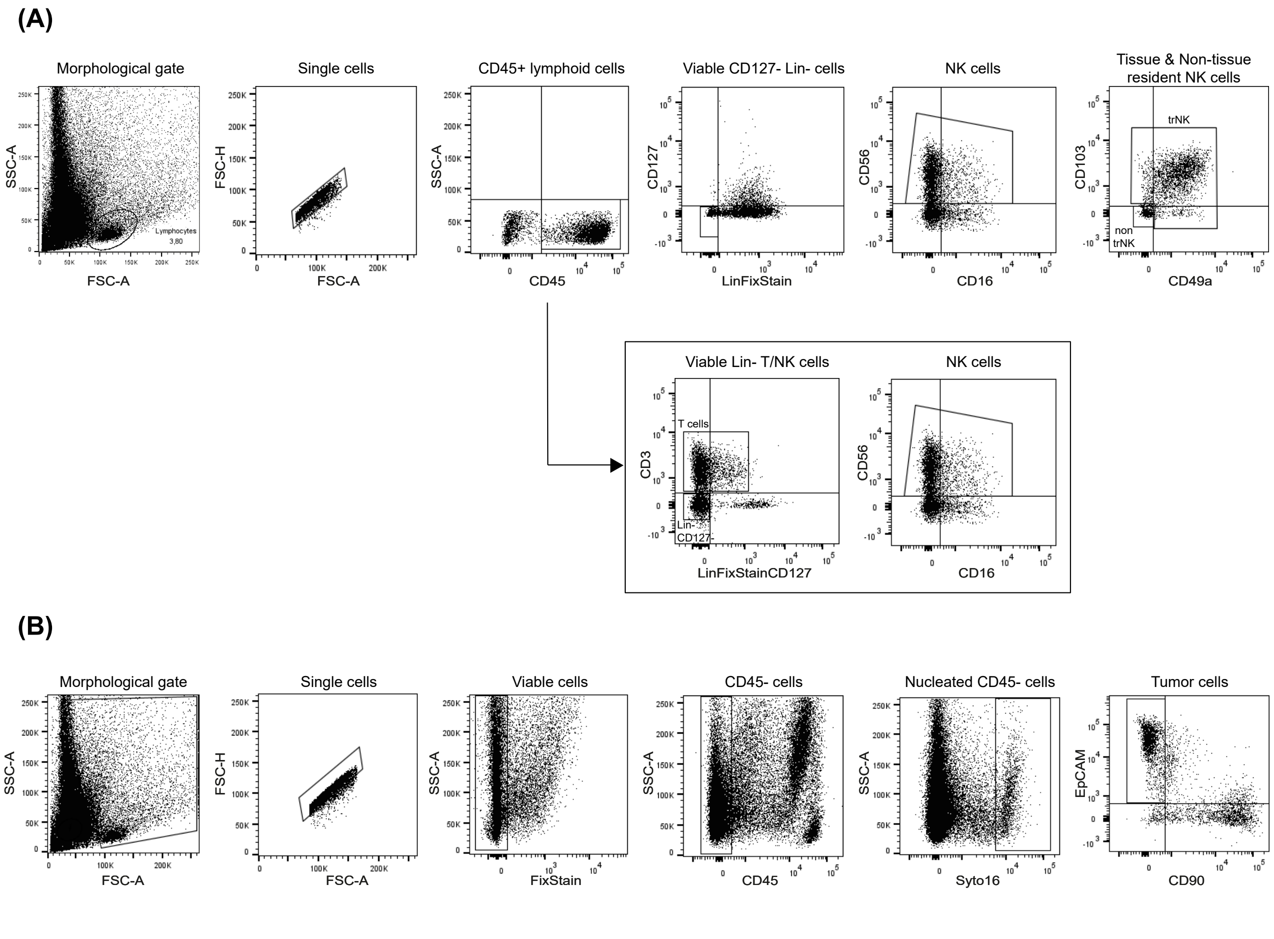

Figure S1. Gating strategies for NK, trNK, T and tumor cells

(A) The gating strategy used to identify NK cells in PB (peripheral blood), T (tumor), TFT (tumor-

free tissue) derived from CRC patients is shown in a representative CRC tumor sample. NK cells were selected first by physical parameters as lymphoid cells (morphological gate) and excluding doublets (single cells), then were identified as $CD45^{+}$, viable, $CD127^{-}Lineage^{-}$ (CD3, CD19, CD14, CD33), $CD56^{+}$ $CD16^{+/-}$ lymphoid cells. Tissue resident NK cells (trNK cells) were subsequently identified by staining with anti-CD49a and anti-CD103 mAbs as $CD49a^{+}CD103^{-}$ or $CD49^{+}CD103^{+}$ or $CD49a^{-}CD103^{+}$ NK cells. For T cell identification and intracellular molecules/activating receptors staining on NK cells, the gating strategy used is shown in the box below. T cells were gated as $CD45^{+}$, viable, $Lineage^{-}$ (CD19, CD14, CD33) $CD127^{+/-}$ $CD3^{+}$ and NK cells as $CD45^{+}$, viable, $CD3^{-}$ $Lineage^{-}$ (CD19, CD14, CD33, CD127 in the same fluorescence channel) $CD56^{+}$ $CD16^{+/-}$ lymphoid cells. (**B)** The gating strategy used to select tumor cells derived from CRC patients is shown in a representative CRC tumor sample. Tumor cells were selected first by physical parameters (morphological gate) and excluding doublets (single cells) and then identified as viable, $Lineage^{-}$ (CD127, CD3, CD19, CD14, CD33, CD56), $CD45^{-}$, nucleated cells ($SYTO16^{+}$), $EpCAM^{+}CD90^{-}$ cells.

Supplementary figure 2

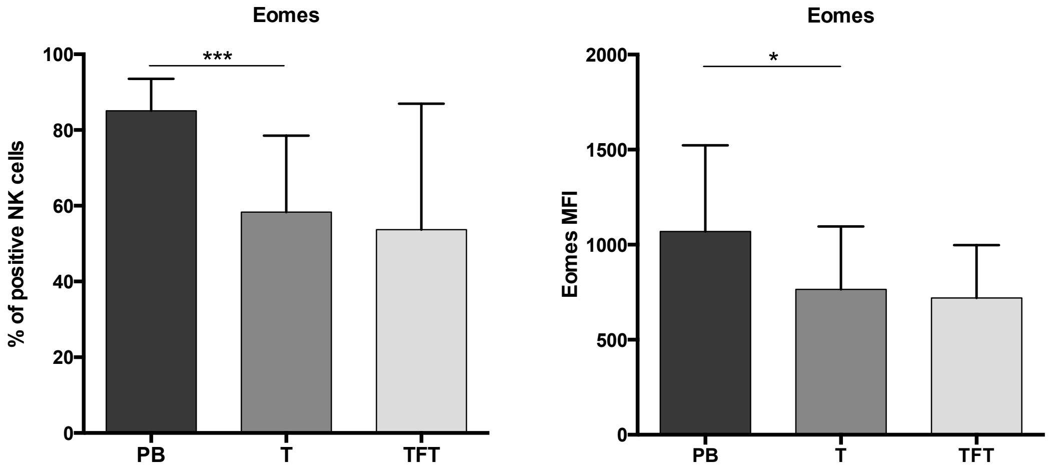

Figure S2. Eomes is expressed by PB-, T- and TFT-NK cells from CRC patients

Intracellular expression of Eomes transcription factor in PB (n=12), T (n=14) and TFT (n=3) - derived NK cells from CRC patients is shown as percentage of positive cells (left) and median fluorescence intensity (MFI) (right). Bars indicate the SD on the mean. Statistical significance computed through Kruskal-Wallis test is indicated (* p<0.05, *** p<0.001).

Supplementary figure 3

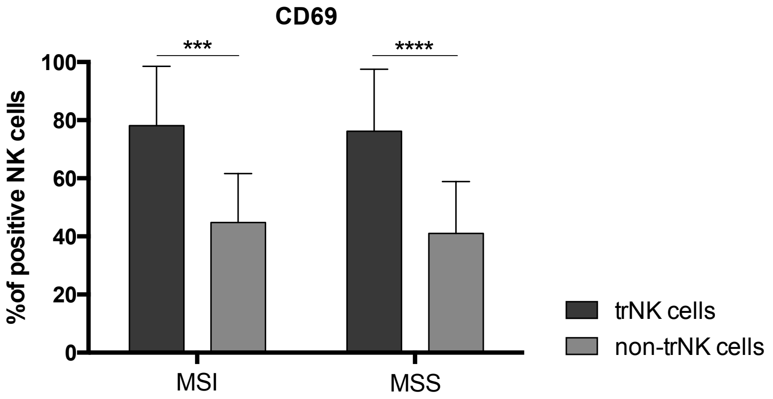

Figure S3. CD69 is expressed by both tumor-associated trNK and non-trNK cells in MSI/MSS CRC patients

The frequency of CD69^{+} tissue resident NK cells (trNK cells) and non-tissue resident NK cells (non-trNK cells) derived from tumor tissue of CRC patients stratified by MMR status (MSI/MSS) is shown (MSI: tr n=14, non-tr n=11. MSS: tr n=31, non-tr n=33). Statistical significance calculated with Mann-Whitney test is indicated (*** $p<0.001$; **** $p<0.0001$).

Supplementary figure 4

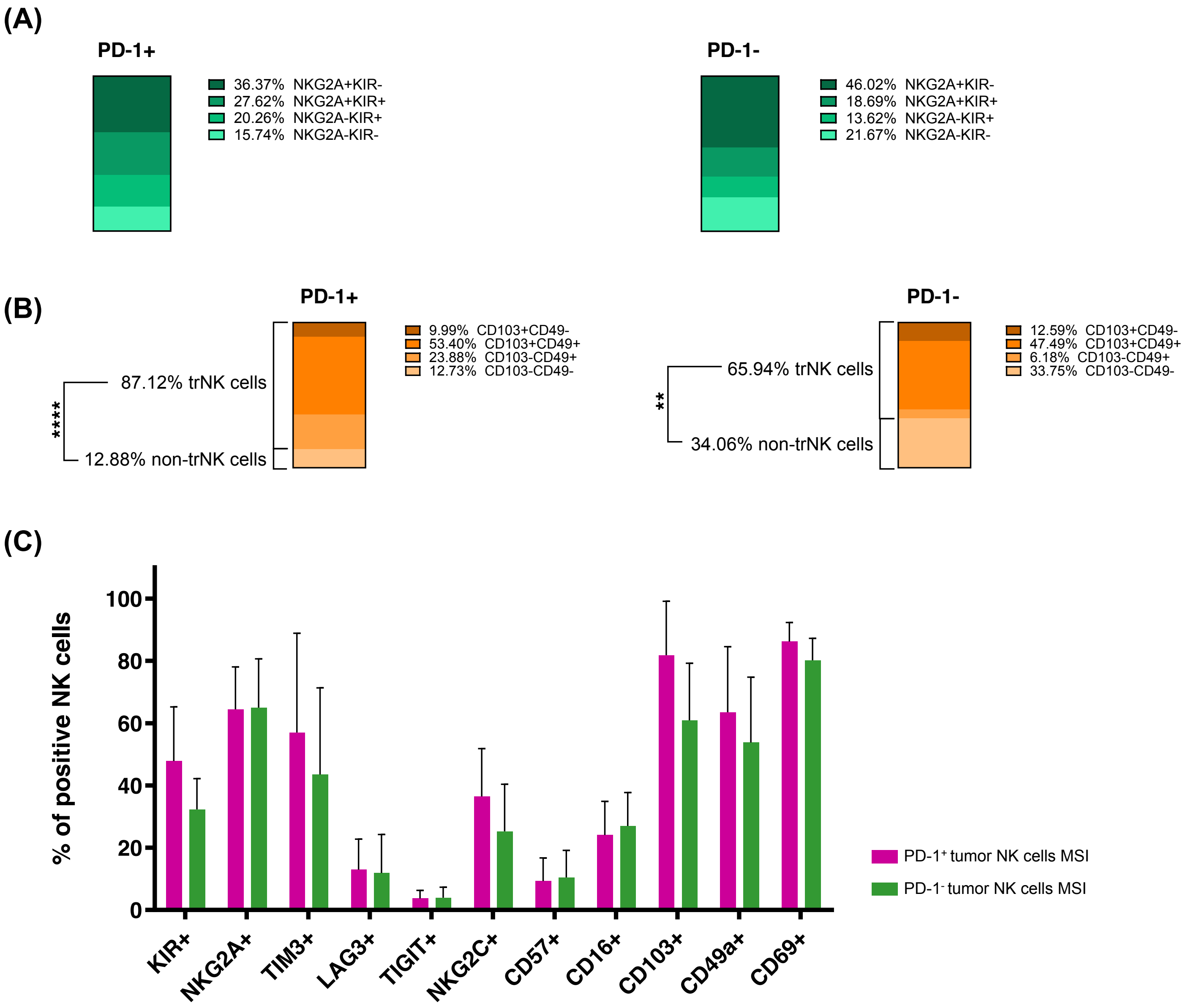

Figure S4. Surface characterization of PD-1$^+$ vs PD-1$^-$ NK cells infiltrating MSI CRC samples **(A)** Percentage of the different NKG2A/KIR subsets expressed on PD-1$^+$ and PD-1$^-$ tumor-associated NK cells in indicated for n=6 MSI CRC patients. No statistically significant difference was found. **(B)** Frequencies of tr- and non-trNK cells and of the different CD103/CD49a subsets are depicted on the stacked bar chart for PD-1$^+$ and PD-1$^-$ tumor-associated NK cells of MSI CRC patients (n=6). Statistical significance calculated by multiple Mann-Whitney test is indicated (* $p<0.05$; ** $p<0.01$). **(C)** Surface expression of the indicated receptors and adhesion molecules (CD103, CD49a, CD69) is shown on PD-1$^+$ (purple bars) and PD-1$^-$ (green bars) tumor-associated NK cells of MSI CRC patients (n=6). Histogram bars represent the mean±SD. No significant difference was found.

Supplementary figure 5

(A)

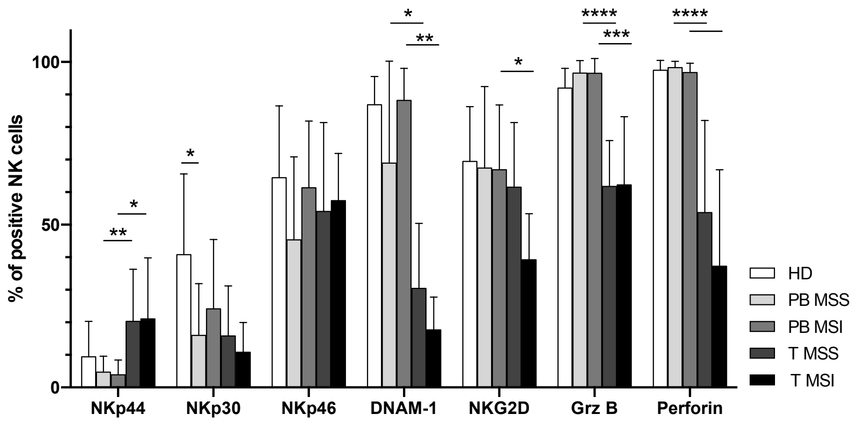

(B)

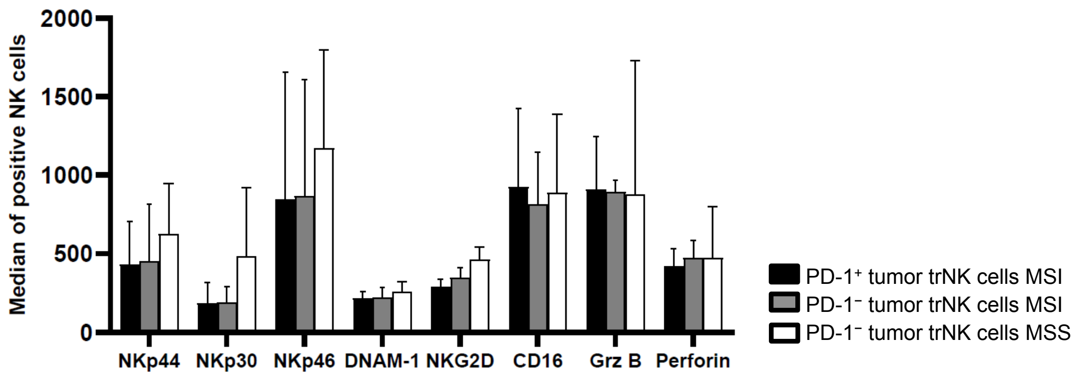

Figure S5. Expression of activating receptors and cytotoxic molecules on NK cells from peripheral blood, tumor and tumor-free tissues in CRC patients

(A) Surface expression of NK activating receptors (NKp44, NKp30, NKp46, DNAM-1, NKG2D) and intracellular expression of granzyme B (Grz B) and perforin were evaluated in healthy donors' PB-NK cells (HD), CRC patients' PB and T -associated NK cells, stratified according to the MMR status (MSI/MSS) (HD n=14, PB MSS n=11, PB MSI n=9, T MSS n=10, T MSI n=9). Bars indicate the SD on the mean. Statistical significance computed through Mann-Whitney test is indicated (*p<0.05; ** p<0.01; *** p<0.001; **** p<0.0001). **(B)** Expression of the indicated activating receptors (actR) (NKp44, NKp30, NKp46, DNAM-1, NKG2D, CD16) and intracellular cytotoxic molecules (intra) (Grz B and perforin) is indicated as median fluorescence intensity on PD-1$^+$ and PD-1$^-$ tumor trNK cells of MSI CRC patients in comparison to PD-1$^-$ tumor trNK cells of MSS CRC patients. Bars indicate the SD on the mean. No statistical significance was found. (actR MSI n=4, actR MSS n=7, intra MSI n=7, intra MSS n=7)

Supplementary figure 6

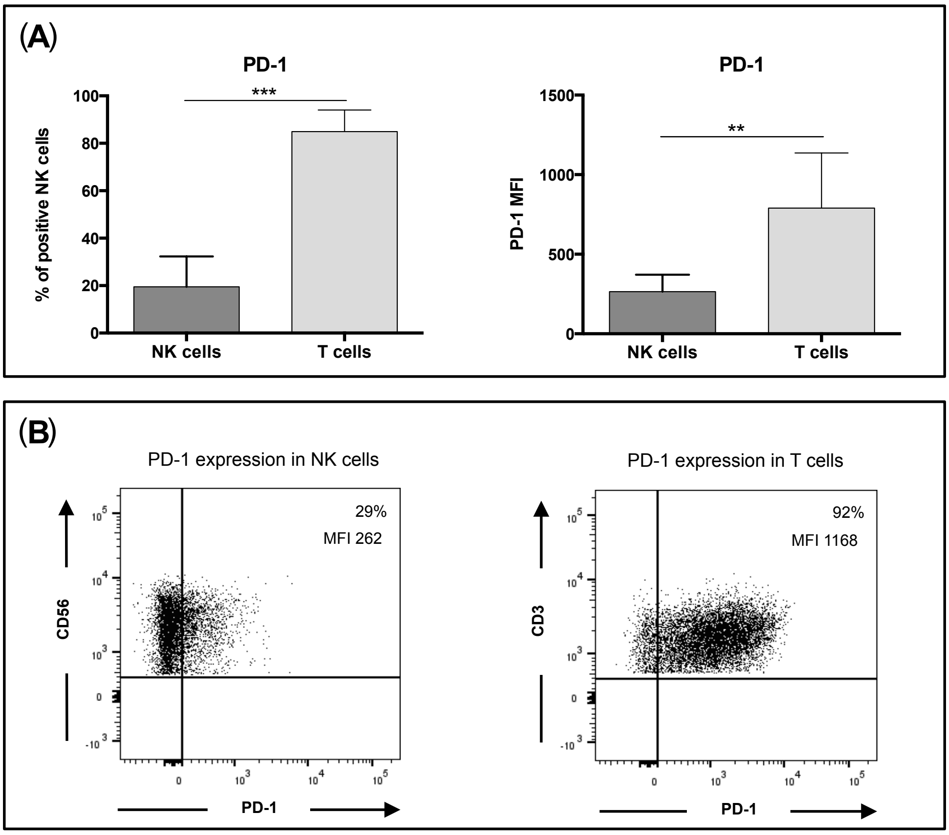

Figure S6. PD-1 expression on tumor-associated NK and T cells from MSI-CRC patients

(A) PD-1 expression is shown as percentage of positive cells (left) and median fluorescence intensity (MFI) (right) for tumor-associated NK cells in comparison to tumor-associated T cells isolated from the same MSI CRC patients (n=7). Histogram bars show the mean and SD. Statistical significances analyzed by Mann-Whitney test are indicated (** $p<0.01$, *** $p<0.001$). (**B)** PD-1 expression on tumor-associated NK (left) and T cells (right) is shown for a representative MSI CRC patient. Percentages of positive cells and MFI are indicated in the upper right quadrant.

Supplementary figure 7

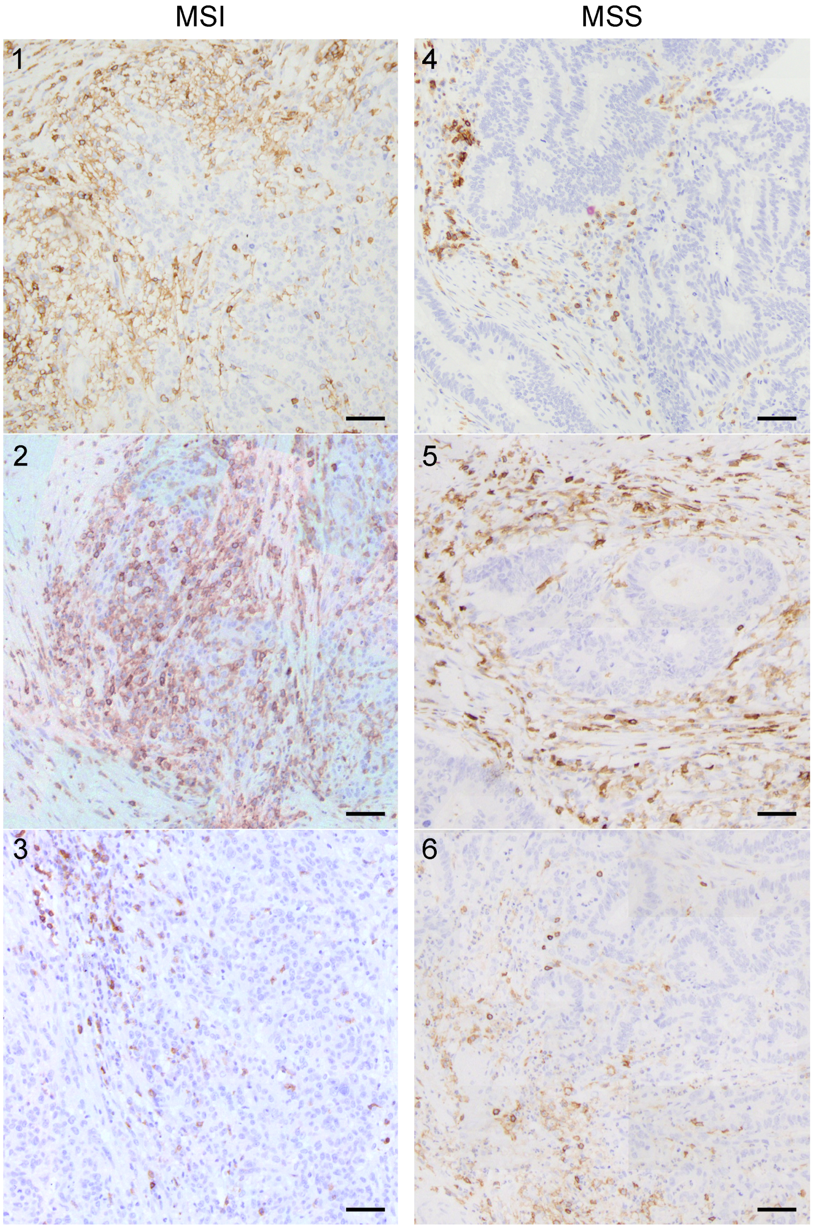

Figure S7. Lymphocytic infiltrate in CRC tissue sections demonstrated by CD45 IHC

CD45 IHC staining is shown on tissue samples corresponding to that depicted in Figure 6. 1-3 show sections from MSI CRC patients; 4-6 show sections from MSS CRC patients. Scale bars are 50 µm. Magnification: 20X.

2.2 Supplementary tables

Table S1 Flow cytometry reagents

Monoclonal antibody	Fluorochrome	Source
CD16-IgG1	Non-conjugated	Produced in our laboratory
DNAM-1-IgG1	Non-conjugated	Produced in our laboratory
KIR3DL1/L2/S1-IgG2a	Non-conjugated	Produced in our laboratory
PD-1-IgG2b	PE or non-conjugated	Miltenyi Biotec, Bergisch Gladbach, Germany
Goat Anti-Mouse IgG1	Alexa Fluor 647	Invitrogen, ThermoFisher Scientific, Waltham, Massachusetts, USA
Goat Anti-Mouse IgG1	BV421	Jackson Immunoresearch, West Grove, Pennsylvania, USA
Goat Anti-Mouse IgG2a	FITC	Southern Biotech
Goat Anti-Mouse IgG2b	PE	Southern Biotech
Goat Anti-Mouse IgG2b	BV421	Jackson Immunoresearch
CD3	APC-R700/PE-CF594/ PerCP-Cy5.5	BD Biosciences, Franklin Lakes, New Jersey, USA
CD14	PE-CF594	BD Biosciences
CD14	Viogreen	Miltenyi Biotec
CD16	APC-H7	BD Biosciences
CD19	PE-CF594	BD Biosciences
CD19	Vioblue	Miltenyi Biotec
CD33	PE-CF594	BD Biosciences
CD45	APC/BUV496	BD Biosciences
CD56	BV480	BD Biosciences
CD56	PE-Cy7	Beckman Coulter, Brea, California, USA
CD57	BUV395	BD Biosciences
CD49a	BV786	BD Biosciences
CD69	BUV737	BD Biosciences
CD85j	APC	Invitrogen, ThermoFisher Scientific
CD90	BV480	BD Biosciences
CD103	BV711	BD Biosciences
CD107a	BB700	BD Biosciences
CD127	BV650/PE-CF594	BD Biosciences
Eomes	PE	Invitrogen, ThermoFisher Scientific
EpCAM	BV786	BD Biosciences
Granzyme B	Alexa Fluor 647	BD Biosciences
HLA-ABC	BV650	BD Biosciences
KIR2DL1/S1	FITC	Miltenyi Biotec
KIRDL2/L3/S2	FITC	BD Biosciences
LAG-3	APC-R700	BD Biosciences
NKG2A	PE-Cy7	Beckman Coulter
NKG2C	BV421	BD Biosciences
NKG2D	BB700	BD Biosciences
NKp30	BV605	BD Biosciences
NKp44	BV650	BD Biosciences
NKp46	BV421	BD Biosciences
PD-L1	APC-R700	BD Biosciences
PD-L2	APC-R700	BD Biosciences
Perforin	PerCP-Cy5.5	BD Biosciences
TIGIT	BV605	BD Biosciences
TIM-3	BB700	BD Biosciences
